# Bacterioplankton Associated with Toxic Cyanobacteria Promote *Pisum sativum* (Pea) Growth and Nutritional Value through Positive Interactions

**DOI:** 10.3390/microorganisms10081511

**Published:** 2022-07-26

**Authors:** Richard Mugani, Fatima El Khalloufi, El Mahdi Redouane, Mohammed Haida, Soukaina El Amrani Zerrifi, Alexandre Campos, Minoru Kasada, Jason Woodhouse, Hans-Peter Grossart, Vitor Vasconcelos, Brahim Oudra

**Affiliations:** 1 Water, Biodiversity and Climate Change Laboratory, Faculty of Sciences Semlalia, Cadi Ayyad University, Av. Prince My Abdellah, P.O. Box 2390, Marrakech 40000, Morocco; richardmugani@gmail.com (R.M.); redouane.elmahdii@gmail.com (E.M.R.); mohammed.haida11@gmail.com (M.H.); soukainaelamranizerrifi@gmail.com (S.E.A.Z.); oudra@uca.ac.ma (B.O.); 2 Department of Plankton and Microbial Ecology, Leibniz-Institute of Freshwater Ecology and Inland Fisheries (IGB), Zur alten Fischerhuette 2, 14775 Stechlin, Germany; minoru.kasada@igb-berlin.de (M.K.); jason.woodhouse@igb-berlin.de (J.W.); hanspeter.grossart@igb-berlin.de (H.-P.G.); 3 Natural Resources Engineering and Environmental Impacts Team, Multidisciplinary Research and Innovation Laboratory, Polydisciplinary Faculty of Khouribga, Sultan Moulay Slimane University of Beni Mellal, P.O. Box 145, Khouribga 25000, Morocco; elkhalloufi.f@gmail.com; 4 CIIMAR, Interdisciplinary Centre of Marine and Environmental Research, Terminal de Cruzeiros do Porto de Leixões, Av. General Norton de Matos, s/n, 4450-208 Porto, Portugal; acampos@ciimar.up.pt; 5 Graduate School of Life Sciences, Tohoku University, Aoba, Sendai 980-8578, Japan; 6 Institute for Biochemistry and Biology, University of Potsdam, Maulbeeralle 2, 14469 Potsdam, Germany; 7 Department of Biology, Faculty of Sciences, University of Porto, Rua do Campo Alegre, 4169-007 Porto, Portugal

**Keywords:** toxic cyanobacterial bloom, microcystins (MC), plant growth-promoting bacteria (PGPB), bacterioplankton, rhizobacteria, oxidative stress, nutritional value

## Abstract

Research on Plant Growth-Promoting Bacteria (PGPB) has focused much more on rhizospheric bacteria. However, PGPB associated with toxic cyanobacterial bloom (TCB) could enter the rhizosphere through irrigation water, helping plants such as *Pisum sativum* L. (pea) overcome oxidative stress induced by microcystin (MC) and improve plant growth and nutritional value. This study aimed to isolate bacteria associated with toxic cyanobacteria, test PGPB properties, and inoculate them as a consortium to pea seedlings irrigated with MC to investigate their role in plant protection as well as in improving growth and nutritional value. Two bacterioplankton isolates and one rhizosphere isolate were isolated and purified on a mineral salt medium supplemented with 1000 μg/L MC and identified via their 16S rRNA gene. The mixed strains were inoculated to pea seedlings in pots irrigated with 0, 50, and 100 μg/L MC. We measured the morphological and physiological parameters of pea plants at maturity and evaluated the efficiency of the plant’s enzymatic and non-enzymatic antioxidant responses to assess the role and contribution of PGPB. Both bacterioplankton isolates were identified as *Starkeya* sp., and the rhizobacterium was identified as *Brevundimonas aurantiaca*. MC addition significantly (*p* < 0.05) reduced all the growth parameters of the pea, i.e., total chlorophyll content, leaf quantum yield, stomatal conductance, carotenoids, and polyphenol contents, in an MC concentration-dependent manner, while bacterial presence positively affected all the measured parameters. In the MC treatment, the levels of the pea’s antioxidant traits, including SOD, CAT, POD, PPO, GST, and ascorbic acid, were increased in the sterile pots. In contrast, these levels were reduced with double and triple PGPB addition. Additionally, nutritional values such as sugars, proteins, and minerals (Ca and K) in pea fruits were reduced under MC exposure but increased with PGPB addition. Overall, in the presence of MC, PGPB seem to positively interact with pea plants and thus may constitute a natural alternative for soil fertilization when irrigated with cyanotoxin-contaminated water, increasing the yield and nutritional value of crops.

## 1. Introduction

The worldwide spread of toxic cyanobacterial blooms has become a global threat to freshwater resources due to the production of cyanotoxins [1,2,3]. One of the major cyanotoxins is microcystin (MC), and MC-rich waters are frequently used for crop irrigation and human/livestock consumption and, thus, reach the food chain [4,5,6]. MC causes serious health problems such as liver cancer, colorectal cancer [7], and malnutrition via nutrient deficiency in crops irrigated with MC-contaminated water.

Freshwaters are most affected by prolonged toxic cyanobacterial bloom events containing MC concentrations ranging from 100 to 10,000 μg/L [8,9,10,11], while most MC concentrations remain below 50 μg MC/L in freshwater [12]. In the Moroccan Lalla Takerkoust lake reservoir, MC-contaminated water is frequently used for the irrigation of economic crops and the generation of drinking water, in which the recorded MC content reached 95.4 μg MC/L in December 2005 [13].

The deleterious effects of MC on plants have been extensively documented in several studies and include growth reduction [14,15], tissue necrosis [9,16,17], photosynthetic machinery inhibition [16,18,19], altered physiological functions, and metabolic disturbances [19,20,21], resulting in altered crop productivity and nutritional quality [18] and subsequent economic losses [2].

The chronic exposure of crops to MC induces oxidative stress by generating excessive reactive oxygen species (ROS) [22,23]. ROS target amino acid residues, degrade proteins and chlorophyll, and destroy chloroplasts [24]. The lipid peroxidation destabilizes membrane permeability and causes significant electrolyte losses [20,21,25].

As a response to oxidative stress, plants activate their antioxidant enzymatic defense system, resulting in an increased production of catalases (CAT), glutathione transferases (GST) [26,27,28], polyphenol oxidase (PPO), peroxidase (POD) [21], superoxide dismutase (SOD) [17,20,25,29], and non-enzymatic ROS-protective compounds, i.e., Polyphenols, Carotenoids [27,28,30], and Ascorbic acid (AsA) [31].

In addition, plants benefit from beneficial relationships with bacteria, which can protect them against ROS-induced oxidative stress. These bacteria are called plant growth-promoting bacteria (PGPB) because they can promote plant growth or yield by improving the plant’s mineral uptake or the availability of essential macro-elements, e.g., phosphorus and potassium [32,33,34]. As a result, PGPB increase plant growth vigor and nutritional value [33,35]. Furthermore, other vital aspects of PGPB include the production of plant growth hormones, such as indole acetic acid (IAA) [34], gibberellins [36,37], exopolysaccharides, and siderophores, and atmospheric nitrogen fixation [38,39].

Studies on PGPB activity during MC-induced ROS stress are scarce. Yet, some studies have shown that plants grown in non-sterile soil and irrigated with MC-contaminated water were protected by their microbiota compared to plants grown on sterile or soilless substrates [40,41]. It has been shown that MC-tolerant rhizobia shielded the plants from oxidative stress and, thus, allowed for effective plant growth [29,42,43]. However, no study has considered the advantage of inoculating plants with consortia of different bacterial species to generate an optimal composition for protecting and optimizing plant growth and yield during irrigation with potentially MC-contaminated waters. Additionally, the diversity of the microbial consortia potentially enhances the plant’s ability to escape toxigenic stress.

In this study, we demonstrated that bacterioplankton isolated from toxic cyanobacterial blooms could (a) tolerate and grow at high MC concentrations, (b) promote plant growth and protect it against ROS-induced oxidative stress, and (c) improve the grain yield and nutritional value of peas after chronic exposure to MC, simulating environmentally relevant doses of MC in the Moroccan Lalla Takerkoust reservoir.

## 2. Materials and Methods

### 2.1. Microcystin Determination 

Cyanotoxins were extracted from a freeze-dried cyanobacterial bloom (dominated by *Microcystis aeruginosa*) collected in October 2010 from the Lalla Takerkoust Reservoir in Marrakesh, Morocco. The extraction process was performed as described in El Khalloufi et al. (2013) [29]. The cyanotoxin extract was passed through C18 solid-phase extraction cartridges (LiChrolut^®^ RP-18, 1000 mg/6 mL, Sigma-Aldrich, Munich, Germany). Following this step, the MCs were eluted with pure methanol, vacuum-dried in rotavapor (40 °C), resuspended in ultrapure water, and stored at −20 °C. The total MC in the cyanobacterial bloom extract used in this study were quantified using the protein phosphatase 2A inhibition (PP2A) assay according to Bouaïcha et al. (2001) [44]. PPA2 analysis revealed a total concentration of 11.5 mg equivalent MC-LR/g freeze-dried cyanobacterial bloom (El Khalloufi et al., 2013) [29].

### 2.2. Bacterial Strain Isolation and Molecular Characterization

*Microcystis aeruginosa* biomass and surface water were collected on 15 September 2019 in the Lalla Takerkoust lake reservoir. Composite soil samples were collected from a farm in the Lalla Takerkoust region irrigated with MC-containing reservoir water. Fifty mL of cyanobacterial biomass was put in a 10 mL flask and shaken at 140 rpm for 4 h to loosen and homogenize the bacterial particulates firmly embedded in the cyanobacterial colonies. The isolation was done according to Shen et al. (2019) [45] in a mineral salt medium (MSM) composed of 1.6 g of K_2_HPO_4_, 0.4 g of MgSO_4_-7H_2_O, 0.5 g of NaCl, 20 mg of CaCl_2_, and 2.3 mg of FeCl_3_-6H_2_O per 1000 mL of water used. A total of 5 g of soil was added to 50 mL of MSM and shaken at 140 rpm for 4 h. Then, 10 mL of cyanobacterial bulk, sludge, and dam water was transferred to a 50 mL MSM medium containing MC and enriched in the same manner once a week for 3 weeks.

The flasks were kept in agitation at 140 rpm during culturing. At each subculture step, the concentrations of MC were gradually increased to a final concentration of 1 mg MC/L. Subsequently, 10-fold serial dilutions of the domesticated bacterial cultures were spread on MSM 1.5% agar plates containing 1 mg MC/L as the sole source of nitrogen and carbon and incubated at 30 °C for 7 days. Individual colonies were tested for their ability to tolerate and grow in liquid MC-enriched MSM, and the optical densities were plotted. The isolates that grew best were named SLB1, SLB3, and RTC10 for bloom-associated, water-related, and soil-related bacterial strains, respectively.

The three isolates were processed by Macrogen Europe B.V. (Amsterdam, The Netherlands), including genomic DNA extraction, PCR amplification, purification, and sequencing using Sanger sequencing. The taxonomic identity of each strain was confirmed by partial sequencing of the 16S rRNA gene. PCR was performed using primer pairs 27F (5′-AGAGTTTGATCCTGGCTCAG-3′) and 1492R (5′-TACGGCTACCTTGTTACGACTT-3′). The thermal cycling conditions were as follows: denaturation of the target DNA at 98 °C for 3 min, followed by 30 cycles at 94 °C for 1 min, primer annealing at 52 °C for 1 min, and primer extension at 72 °C for 5 min. At the end of the cycle, the reaction mixture was held at 72 °C for 5 min and then cooled to 4 °C.

The sequencing was performed using primer pairs 785F (5′-GGATTAGATACCCTGGTA-3′) and 907R (5′-CCGTCAATTCMTTTRAGTTT-3′). The isolates’ partial 16S rRNA gene sequences were deposited in GenBank (NCBI, Bethesda, MD, USA) under the accession numbers OM754556, OM754557, and OM754558 for SLB3, SLB1, and RTC10, respectively. A total of 16S rRNA sequences and those of representative sequences from GenBank were aligned using ClustalW, and non-overlapping terminal regions were removed. The final alignment comprised 1447 nucleotide positions (an average of 1336 nucleotides for each sequence). *Escherichia coli* (NR_024570.1) and *Pseudomonas aeruginosa* (NR_026078.1) sequences were included as outgroups. Phylogenetic tree reconstruction was performed using PhyML version 3.0 (ATGC Montpellier Bioinformatics, Montpellier, France) with the HKY85 model of nucleic acid substitution and a bootstrap re-sampling value of 1000. Bootstrap values were converted to percentages, and bootstrap values less than 50% were not annotated. 

### 2.3. Growth-Promoting Properties Test of Isolates 

#### 2.3.1. Phosphate Solubilization

##### Qualitative Estimation of Phosphate Solubilization in an Agar Medium

The estimation of phosphorus solubilization for the three isolates was tested on theNational Botanical Research Institute’s phosphate growth medium devoid of yeast extract (NBRIY) and composed of 10 g glucose, 5 g Ca_3_(PO_4_)_2_, 0.5 g (NH_4_)_2_SO_4_, 0.2 g NaCl, 0.1 g MgSO_4_·7H2O, 0.2 g KCl, 0.002 g MnSO_4_·H_2_O, 0.002 g FeSO_4_·7H_2_O, and 15 g agar in distilled water according to Alikhani et al. (2006) [46] and Nautiyal et al. (1999) [47]. After 10 days of incubation, the solubilization index (SI) was measured using the following formula: SI = diameter of the solubilization halo/diameter of the colony [32,48] 

##### Quantitative Estimation of Phosphate Solubilization in a Liquid Medium

To determine the amount of phosphorus solubilized in the NBRIY liquid medium, isolates were prepared according to Bechtaoui et al. (2019) [32]. The colorimetric approach based on the reduction of a phosphorus-molybdate complex was used to determine soluble phosphate [49].

#### 2.3.2. Qualitative Estimation of Potassium Solubilization in an Agar Medium

Potassium solubilization was estimated according to Alikhani et al. (2006) [46] using Alexandrov’s solid medium composed of 5 g glucose, 0.5 g, MgSO_4_. 7H_2_O, 0.1 g CaCO_3_, 0.006 g FeCl_3_, 2 g Ca_3_PO_4_, 3 g insoluble mica powder as a potassium source, and 15 g agar in distilled water, according to Parmar and Sindhu (2013) [50]. After 7 days, the colony diameters and their halos were measured, and the solubilization index (SI) was expressed as the ratio of the halo diameter to the colony diameter (DH/DC).

#### 2.3.3. Exopolysaccharide Production

The isolates’ ability to produce exopolysaccharides (EPS) was assessed according to Spiers et al. (2003) [51] and Lee et al. (2007) [52]. The EPS produced were expressed as the mg of congo red bound to exopolysaccharide divided by the bacterial optical density measured at 600 nm (mg of CR/OD_600_).

#### 2.3.4. Phytohormone Production: Indole-3-Acetic Acid (IAA)

The phytohormone production by each isolate was determined on Luria-Bertani (LB) broth containing 0.5 mM tryptophan as a precursor of indole acetic acid and incubated at 28 °C for 4 days. The assay was performed according to Rahman et al. (2010) [53] and Bano & Musarrat (2003) [54]. The amount of auxin produced was calculated using a sodium indole-3-acetic acid standard curve with different concentrations from 10 to 60 μg/mL. 

### 2.4. Plant Material and Experimental Design

New seeds (2020 harvest) of the Lincoln pea cultivar (*Pisum sativum*) were purchased from Agrival Marrakech. Healthy and homogeneous seeds were selected, and their surface was sterilized with sodium hypochlorite (6%) for 10 min.

The seeds were thoroughly rinsed with sterile distilled water and germinated at 22 °C in the dark in sterile glass Petri dishes lined with filter paper lightly soaked with sterile distilled water. After germination, the seedlings were selected for uniformity, and some were inoculated with a bacterial suspension (in physiological water) of the isolates SLB1, SLB3, and RTC10 and sequentially labeled A, B, and C, respectively, to simplify subsequent notations. Some germinated pea seedlings were inoculated with the bacterial consortia, and some other sterile seedlings were kept for control (NI). The inoculated and sterile seedlings were planted in pots—three seedlings per pot—on 6 December 2020 in eight pots per treatment (*n* = 24 seedlings per addition). The pots contained agricultural soil sterilized at 200 °C for 4 h in a muffle furnace. The physicochemical characteristics of the soil and well water used are shown in Table 1. 

Chronic exposure to MC began 10 days after the germinated pea seedlings were transplanted into the pots. Plants were grown for 12 weeks between 6 December 2020 and 4 March 2021 in a greenhouse in Marrakech under natural photoperiod and temperature conditions.

For MC treatment, all pots were divided into three experimental groups. The first group was irrigated with MC-free water (control group). In contrast, the two other groups were irrigated with water spiked with an equivalent of 50 and 100 μg MC/L, respectively. The MC used to contaminate the water were previously purified on a C18 solid-phase extraction cartridge (LiChrolut^®^ RP-18, 1000 mg/6 mL, Sigma-Aldrich, Munich, Germany) and filtered on 0.22 µm Glass Fiber and 25 mm Luer-Lok/Luer Slip. These concentrations reflect the natural conditions for MC quantified in the Lalla Takerkoust reservoir during the cyanobacteria bloom period [13].

**Figure 1:**
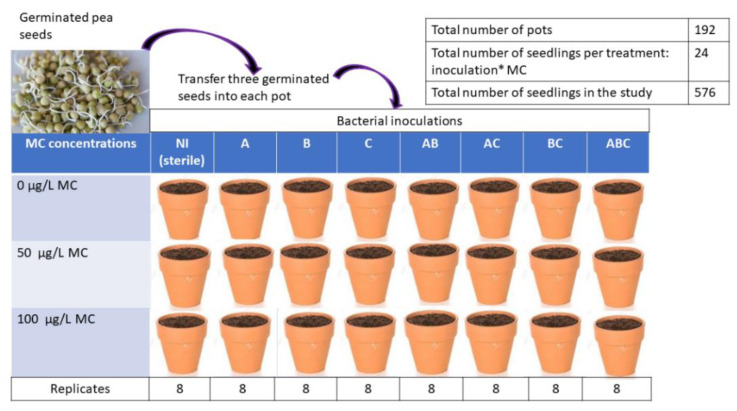
Experimental design for pea bacterial addition and MC treatment.

For bacterial addition, the pots were divided into eight experimental groups, including NI (sterile-control), A, B, C, A × B, A × C, B × C, and A × B × C, corresponding to the different combinations of isolates: SLB1 = A, SLB3 = B, and RTC10 = C. Each treatment comprised three seedlings per pot and was repeated eight times, making a total of 192 pots and 576 seedlings (Figure 1). The physicochemical properties of the soil and water used in this study are shown in Table S1.

### 2.5. Assessment of Pea Physiological Responses

Stomatal conductance, chlorophyll fluorescence, and chlorophyll pigment concentration were used to examine plant physiology. To determine chlorophyll fluorescence (Fv/Fm) and stomatal conductance (gs), fully expanded leaves were examined. Fv/Fm was measured using a modulated chlorophyll fluorometer (model OSI 30p, Optisciences). A porometer (leaf porometer, model SC1) was used to assess stomatal conductivity. Photosynthetic pigments (chlorophylls and carotenoids) in leaves were determined according to Upadhyay and Pame (2019) [55]. The amounts of chlorophyll a, chlorophyll b, and carotenoids were determined using a UV-visible spectrophotometer to measure optical densities at 662, 644, and 470 nm (Cary 50 Scan, Melbourne, Australia). Total chlorophyll (a + b) and carotenoids (Car) were expressed as mg/g of fresh weight.

### 2.6. Plant Harvest and Sampling

Harvesting was performed at the fruiting stage. Plants were washed and separated into three groups according to MC concentrations (0, 50, and 100 μg/L). Then, each group was divided into eight subgroups according to bacterial additions (NI, A, B, C, A × B, A × C, B × C, A × B × C). The morphological growth and yield parameters that were recorded were stem length (SL), leaves number per plant (LNP), main root length (MRL), and pod number per plant (PNP). Each of the eight subgroups was divided into two parts.

In the first part, the grain number per pod (GNPo) was recorded, and the shoot dry weight (SDW) and root dry weight were determined after drying at 60 °C for 48 h to constant weight. 

In the second part, pods and leaves were separated, immersed in liquid nitrogen, and stored at −20 °C. The frozen material was used to determine pigments, Ascorbic acid (AsA), Polyphenols (GAE), Malondialdehyde (MDA), sugars, and antioxidant enzymes.

Structural integrity: electrolyte leakage (EL) and lipid peroxidation (MDA)

The cellular structural impairments in stressed plants were assessed by analyzing electrolyte leakage (EL), lipid peroxidation, and Malondialdehyde (MDA). Electrolyte leakage (EL) was determined according to Ali et al. (2021) [56]. The EL was expressed as a percentage of electrolyte loss.

The Lipid Peroxidation in leaves was analyzed, as described by Velikova et al. (2000) [57], by estimating thiobarbiturate reactive substances (TBARS). The MDA concentration was expressed as nmol/g fresh weight (FW). 

Analysis of Mineral Nutrients, Total Phosphorus, and Crude Protein

Inorganic ions, Ca^++^, K^+^, and Na^+^ were dosed in fruits using the Sedico Ltd.—AFP-100 Flame Photometer. Another volume was used to determine total phosphorus in the leaves and fruits, according to Olsen & Sommers (1982) [49]. The crude protein content in the leaves and fruits was estimated, according to Mariotti et al. (2008) [58], using the following formula:

CPL/F (g/100 gDW) = %NTK × 5.6

where CPL/F is the total protein concentration in leaves (L) or fruits (F). %NTK is the percentage of total Kjeldahl nitrogen in leaves or fruits. 5.6 is a default conversion factor. DW is the material dry weight

### 2.7. Biochemical Investigation

#### 2.7.1. Determination of Soluble Sugars, Polyphenols, and Ascorbic Acid

The content of soluble sugars in the leaves and fruits was measured according to Dubois et al. (1956) [59]. A hundred mg of fresh material was crushed and homogenized in 10 mL of 80% ethanol and centrifuged at 5000 rpm for 10 min. A total of 200 µL of supernatant was added, and 200 µL of phenol (5%) and 1 mL of concentrated H_2_SO_4_ were added to 200 µL of supernatant. The absorbance was measured at 485 nm in a UV-visible spectrophotometer (Square 50 Scan, Sydney, Australia), and the concentration of soluble sugars was calculated using glucose as the standard.

The total phenolic content expressed as the gallic acid equivalent was determined according to the Folin–Ciocalteu method [60]. A total of 0.1 g of dried leaves were ground to a fine powder and homogenized in 2 mL ethanol 95%. The homogenate was centrifuged at 30,000× *g* for 10 min, and the supernatant was adjusted to 10 mL with distilled water. A total of 1 mL of the solution was mixed with 1 mL of 1 N Folin–Ciocalteau reagent and 1 mL of 10% sodium carbonate, and the mixture of four replicates was incubated for 1 h at 35 °C. The absorbance was measured at 765 nm, and the content of phenolic compounds expressed as the gallic acid equivalent per gram of dry weight was calculated from the gallic acid calibration curve.

Ascorbic acid was determined according to the titrimetric method [61]. A total of 0.5 g of fresh seeds was ground finely in a mortar, recovered with 3 mL of 2% HCl, and allowed to stand for 10 min. The vitamin extract was centrifuged at 5000 rpm at 4 °C for 10 min. Then, 1 mL of the extract and 3 mL of distilled water were added in an Erlenmeyer flask, and three drops of 0.5% starch solution were added as an indicator. The mixture in four replicates was quickly titrated with 0.01 N iodine solution. The endpoint of the titration was identified as the first permanent trace of a dark blue-black solution. The ascorbic acid content was calculated according to the formula:

$$\mathrm{AsA}=\frac{\left(\mathrm{Ve}-\mathrm{Vb}\right)\times \;M\;\times 176.12\times \;\mathrm{Vt}\;\times 100}{P\;\times \;V}$$

where

With AsA = mg of ascorbic acid in 100 g of fresh material
Ve = mL of iodine used to titrate the extract
Vb = mL of iodine used to titrate the blank (control = 2% HCl)
Vt = total volume of the extract
M = concentration of iodine (0.01 M)
P = weight of freshly ground material (yr)
V = volume of the titrated extract.

#### 2.7.2. Oxidative Stress Analysis: Antioxidant Enzymes

The oxidative stress assay was performed on the frozen leaves of plants exposed to MC at different concentrations. Crude enzyme extracts were prepared to measure the activities of glutathione S-transferase (GST, EC 2.5.1.18), catalase (CAT, EC 1.11.1.6), superoxide dismutase (SOD, EC1.15.1.1), polyphenol oxidase (PPO, EC1.14.18.1), and peroxidase (POX, EC 1.11.1.7). Briefly, 500 mg of fresh plant material was ground into phosphate buffer (0.1 M, pH 6.5, 20% glycerol, 1.4 mM 1,4-Dithioerythritol, 1 mM phenylmethylsulfonylfluoride) in an ice bath. Thereafter, the homogenate was centrifuged at 10,000 g at 4 °C for 10 min. The pellet was re-extracted and centrifuged as before. The supernatants (enzyme extracts) were pooled and stored at −20 °C. The protein content of the extract was determined according to Bradford (1976) [62], and the samples were diluted to 0.3 mg/ mL protein before measuring the GST activity. 

The GST activity was determined as described in Habig et al. (1974) [63]: 600 µL of a cocktail containing 0.1 M (6.5) phosphate buffer, 10 mM reduced glutathione, and 60 mM 1-chloro-2,4-dinitrobenzene was added to 300 µL of the enzyme extract. CAT activity was determined as described in Rao and Paliyath (1994) [64]: 1 mL of 0.3% (*v*/*v*) H_2_O_2_ was diluted in 1.9 mL of distilled water and added to 1 mL of the enzyme extract. The catalase activity was monitored by measuring the change in absorbance at 340 nm and 240 nm every 20 s for 3 min for GST and CAT, respectively, using a Varian Cary^®^ 50 UV-Vis spectrophotometer (Agilent Technologies, Santa Clara, CA, USA).

Superoxide dismutase (SOD) activity was determined according to Beyer and Fridovich (1987) [65] by detecting the reduction of nitroblue tetrazolium (NBT) to formazan blue using a microplate reader at 560 nm after 30 min under blue light. The reaction mixture contained 170 µL of phosphate buffer (0.1 mM, pH 6.0), 30 µL of methionine (55 mM), 30 µL of NBT (0.75 mM), and 10 µL of enzyme extract. The reaction was initiated by adding 30 µL of riboflavin (0.1 mM).

The POD activity was measured according to Polle et al. (1994) [66] based on the production of tetragaiacol from the oxidation of guaiacol. The reaction mixture contained 0.1 mL of the enzyme extract, 3 mL of 1 M phosphate buffer (pH 7.0), 1 mL of 20 mM guaiacol, and 0.5 mL of 0.3% H_2_O_2_. The absorbance was read at 470 nm (ε = 25.5 mM cm^−1^) for 90 s (00 s, 60 s, and 90 s). POD activity was expressed per µmol guaiacol oxidized min^−1^ mg^−1^ protein.

PPO activity was determined according to the method described by Wojdyło et al. (2013) [67] based on catechol oxidation. The reaction medium contained 200 μL of the enzyme extract and 1 mL of 10 mM catechol dissolved in 100 mM phosphate buffer (pH 6.4). The absorbance at 410 nm was measured. The PPO activity was expressed as a unit per mg of protein. For all the enzyme activity assays, four replicates were used per sample.

### 2.8. Statistical Analyses and Data Processing

All the statistical analyses were performed with R Studio software, version R.4.1.2 (R Foundation for Statistical Computing, Vienna, Austria, www.r-project.org). All measurements in this study were conducted in four replicates. To highlight the variability of MC effects on the growth and nutritional value of peas, besides the protective role of bacterial addition to this plant, a comparison of medians was performed by the Kruskal–Wallis rank sum test using a significance level of 5% (*p* ≤ 0.05). Wilcoxon pairwise comparisons with multiple test corrections used the Benjamini–Hochberg false discovery rate (BH-FDR) correction. Depending on the level of damage caused to the plant by MC concentration or the gain following the bacterial addition, a decrease or increase in the activity ratio was computed.

A principal component analysis was performed on a correlation matrix of 31 variables (i.e., scale. = TRUE) in order to highlight the suitability of the variables chosen for the study and their contribution to the problem evaluated. The database used was submitted to a factorial analysis adequacy test by the Kaiser–Meyer–Olkin (KMO) test, providing an index tending towards 1 for a perfectly adequate matrix for a factorial analysis. Similarly, the determinant of the correlation matrix was calculated to overcome multicollinearity. Furthermore, as a condition for the principal component analysis, Bartlett’s test of sphericity was performed to detect the minimum correlation between the variables. Except for the heatmap, which was created with R studio, all other figures were achieved with GraphPad^®^ Prism v9.0 (GraphPad Software, San Diego, CA, USA)

## 3. Results

### 3.1. Tolerance Test and PGPB Molecular Characterization

Bacterial strains that can tolerate higher concentrations of MC were used in this study for further experiments. Four strains that exhibited large colonies on the solid MSM medium were tested in an MC-spiked liquid MSM medium for growth ability and MC tolerance. The cultures were incubated at 30 °C and shaken at 140 rpm. After 30 h of culture (Figure S1), the growth assayed by optical density analysis, which showed a highly significant difference between the Kruskal–Wallis test of H (4) = 45.203 (*p* = 0.001) and the Bonferroni correction. While the subgroups SLB1, SLB3, and RTC10 did not differ significantly at the 5% cut-off between pairs, the strain BL10 differed significantly from SLB1 and SLB3. Similarly, the sterile control differed significantly from the four other additions, *p* < 0.001. Thus, the isolates SLB1, SLB3, and RTC10, designated respectively by the letters A, B, and C in the following document, were chosen for the study. 

**Figure 2:**
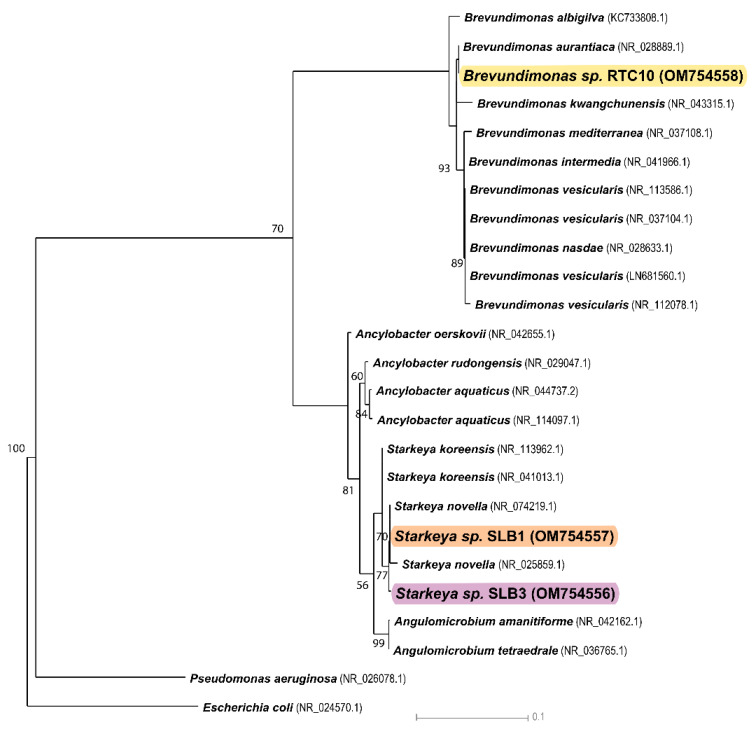
Phylogenetic tree of SLB1, SLB3, and RTC10 strains (highlighted by different colors in figure capture) and their nearest clustering neighbors. Evolutionary history was inferred using the Neighbor-Joining method. The maximum likelihood phylogenetic tree was performed in PhyML version 3.0 with the HKY85 nucleic acid substitution model and a bootstrap resampling value of 1000, using 25 sequences of 1336 nucleotides, on average, for each sequence. Bootstrap values were converted to percentages, and the branches corresponding to the partitions replicated in less than 50% of the bootstrap replicates were collapsed. One *Escherichia coli* sequence (NR_024570.1) and one *Pseudomonas aeruginosa* sequence (NR_026078.1) served as the outgroups.

The 16S rRNA gene sequence and phylogenetic analysis of the three strains of interest (SLB1, SLB3, and RTC10) exhibiting rapid growth in the presence of high concentrations of MCand revealed that the planktonic bacterial strains SLB1 and SLB3 belong to the genus *Starkeya* and are closely related to the species *Starkeya novella*. Conversely, the rhizosphere strain RTC10 belongs to the genus *Brevundimonas* and is related to the species *Brevundimonas aurantiaca* (Figure 2).

### 3.2. Plant Growth-Promoting Properties of Bacteria (PGPB)

Four plant growth-promoting properties of bacterial strains were measured (Table 1). These properties include phosphorus solubilization on both solid and liquid media, potassium solubilization on solid media, and the production of exopolysaccharides and phytohormone indole acetic acid (IAA). For phosphorus solubilization in a solid medium, only the SLB1 strain showed a pronounced halo after 12 days, with a ratio of the halo diameter to the colony diameter DH/DC of 1.84, while the DH/DC of the SLB3 strain was 1. The rhizobacterial strain RTC10 could not grow on the solid NBRIY medium containing tricalcium phosphate. However, in the liquid medium, the strain RTC10 recorded the highest solubilization, which was statistically different from the other strains (*p* < 0.001). Contrarily, strain SLB1 showed the highest solubilization in the solid medium but the lowest solubilization in the liquid medium.

Similarly, the bacterial strains solubilized potassium on a solid Alexandrov medium. The SLB1 strain solubilized more K than the other strains, with a DH/DC of 2.12 ± 0.75. The rhizobacterial strain showed no growth on the same Alexandrov medium. Exopolysaccharide production was mainly carried out by strain SLB1, with 231.07 ± 26.22 μg of CR/OD 600. All the strains produced IAA, whereby strain SLB1 showed the highest production capabilities, with 231.5 ± 2.16 μg/mL. According to the above results, we observed that the planktonic bacteria are the most active in plant growth-promoting properties, in the following order: SLB1 > SLB3 > RTC10.

### 3.3. Physiological Responses: Total Chlorophyll (a + b), Carotenoids, Leaf Quantum Yield, and Stomatal Conductance

**Figure 3:**
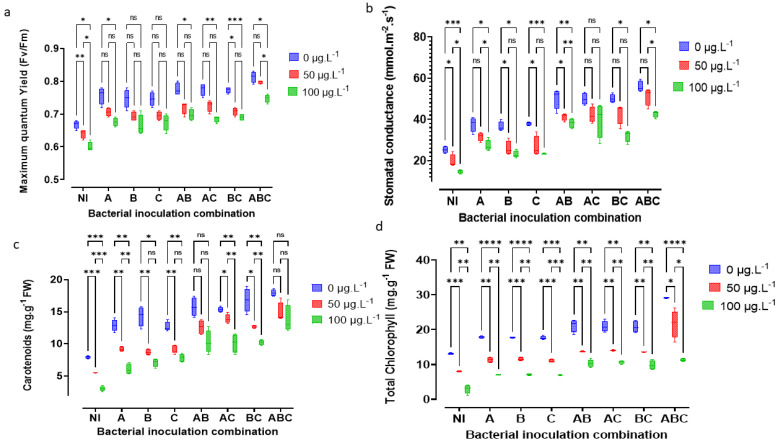
Detrimental impact of MC and beneficial effect of bacterial addition on the maximum quantum yield, MQY, in Fv/Fm (**a**); stomatal conductance, gs, in mmol m^−2^ s^−1^ (**b**); Carotenoids, Car, in mg g^−1^ fresh weight (**c**); and total chlorophyll (a + b), TChl, in mg g^−1^ fresh weight (**d**). The letters NI correspond to the sterile control without bacteria. A = SLB1 is the strain isolated from *Microcystis*-bloom, B = SLB3 is the strain isolated in MC—contaminated water, and C = RTC10 is the strain isolated in agricultural MC contaminated with MC soil. The stars correspond to statistical significance at different levels as ns= not significant; * = *p* < 0.05; ** = *p* < 0.01; *** = *p* < 0.001; **** = *p* < 0.0001.

The pea’s physiological state and the number of photosynthetic pigments are shown in Figure 3. MC concentrations negatively impacted the sterile plants’ physiology and photosynthetic pigments, while bacterial addition had a beneficial effect on the same plant parameters. The effect of MC concentration was highly significant on the maximum quantum yield H(2) = 33.711, *p* < 0.001; stomatal conductance H(2) = 19.296, *p* < 0.001; Carotenoids H(2) = 33.32, *p* < 0.001; and total chlorophyll H(2) = 65.101, *p* < 0.001. Similarly, the recovery advantage provided by bacterial addition was highly significant on the same variables: MQY H(7) = 47.334, *p* < 0.001; gs H(7) = 66.746, *p* < 0.001; car H(7) = 52.423, *p* < 0.001; and TChl H(7) = 25.397, *p* < 0.001.

A post hoc comparison at the 5% threshold using the Benjamini–Hochberg false discovery rate correction for MC shows that all pairwise groups are highly significant, *p* < 0.05. For bacterial addition, highly significant differences were noted between groups differing in terms of addition level. Treatments with the same level of bacterial addition—one strain (A with B or C) or two strains (A × B with A × C or B × C)—were not significantly different. In the sterile control, the concentration of 100 μg MC/L conferred decreases of 10.45% MQY, 77.28% TChl, 41.73% gs, and% 61.64 Car. On the other hand, when comparing 0 and 100 μg MC/L, the triple bacterial addition with SLB1, SLB2, and RTC10 resulted in increases of 20.9% and 11.94% for MQY; an increase of 123.41% and a decrease of 12.97% TChl compared to a decrease of 77.28% in the sterile control; increases of 121.22% and 68.82% gs; and increases of 125.66% and 72.83% Car, respectively.

### 3.4. Morphometric and Yield Indicators

**Figure 4:**
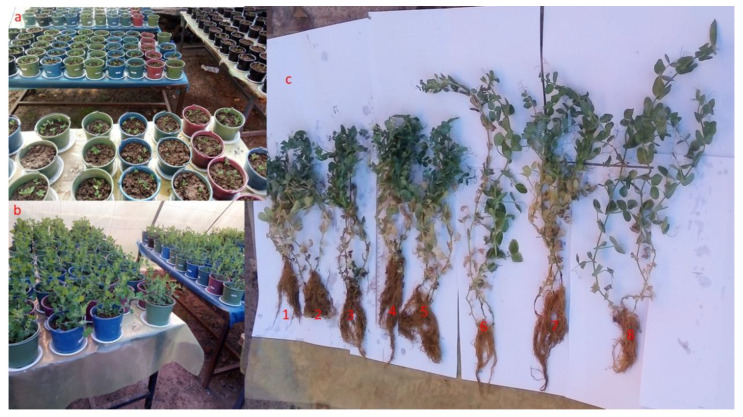
Morphometric appearance of pea plants (**a**) at the time of seedling transplantation into pots, (**b**) at the vegetative stage, and (**c**) after three months of cultivation at the time of harvest (without fruits); in (**c**), plants watered with 100 µg MC/L and the bacterial strains were added in different combinations, where 1, 2, 3, 4, 5, 6, 7, and 8 correspond to NI (sterile control) and inoculation with A, B, C, A × B, A × C, B × C, and A × B × C respectively.

**Figure 5:**
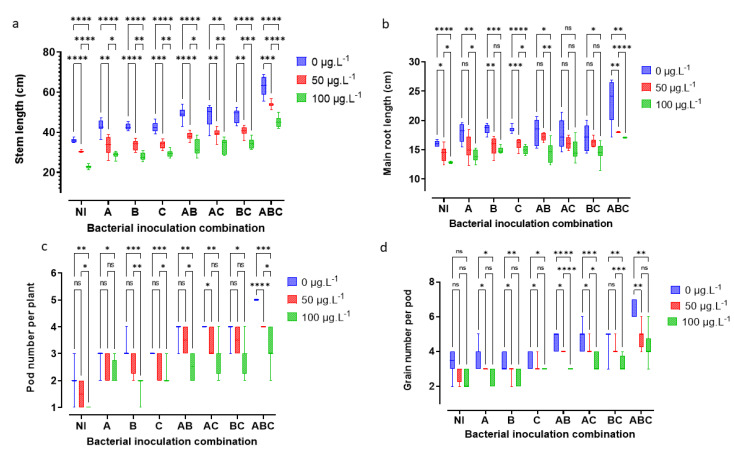
Detrimental impact of MC and beneficial effects of bacterial addition on stem length, SL, in cm (**a**); main root length, MRL, in cm (**b**); number of pods per plant, PNP, (**c**); and number of grains per pod, GNPo, (**d**). The letters NI correspond to the sterile control without bacteria. A = SLB1 is the strain isolated from the *Microcystis* bloom, B = SLB3 is the strain isolated in MC-contaminated water, and C = RTC10 is the strain isolated in agricultural MC-contaminated soil. The stars correspond to statistical significance at different levels as ns= not significant; * = *p* < 0.05; ** = *p* < 0.01; *** = *p* < 0.001; **** = *p* < 0.0001.

**Figure 6:**
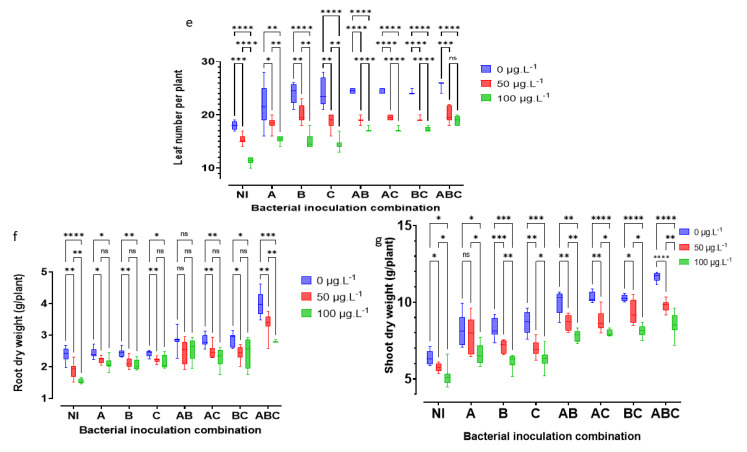
Detrimental impact of MC and beneficial effects of bacterial addition on leaf number per plant, NLP, (**e**); root dry weight, RDW, in g/plant (**f**); and shoot dry weight, SDW, in g/plant (**g**). The letters NI correspond to the sterile control without bacteria. A = SLB1 is the strain isolated from the *Microcystis* bloom, B = SLB3 is the strain isolated in MC-contaminated water, and C = RTC10 is the strain isolated in agricultural MC-contaminated soil. The stars correspond to statistical significance at different levels as ns= not significant; * = *p* < 0.05; ** = *p* < 0.01; *** = *p* < 0.001; **** = *p* < 0.0001.

MC retards plant growth (Figure 4) and thereby reduces yields. Figure 5 and Figure 6 show MC-induced growth inhibition, yield reduction, and the benefits of bacterial-added bacteria, alone or in consortia. The Kruskal–Wallis test shows highly significant differences in the effects of MC of sterile and non-sterile treatments on GNPo H(2) = 19.25, *p* < 0.001; LNP H(2) = 57. 854, *p* < 0.001; MRL H(2) = 35.3, *p* < 0.001; PNP H(2) = 20.16, *p* < 0.001; RDW H(2) = 22.036, *p* < 0.001; SDW H(2) = 22.622 *p* < 0.001; and SL H(2) = 39.767, *p* < 0.001. Additionally, there were highly significant differences for bacterial addition: GNPo H(7) = 46.983, *p* < 0.001; LNP H(7) = 21.474, *p* < 0. 001; MRL H(7) = 24.481, *p* < 0.001; PNP H(7) = 51.056, *p* < 0.001; RDW H(7) = 42.019, *p* < 0.001; SDW H(7) = 56.259, *p* < 0.001; and SL H(7) = 43.177, *p* < 0.001. Plant damage increased with increasing MC concentrations. The concentration of 100 μg MC/L was the most damaging. On the other hand, as the number of bacteria in the consortium increased, the tolerance in the plant to the effects of MC increased (Figure 5). Thus, the bacterial combination A × B × C allowed the plant to combat the negative effects of cyanotoxins. For example, with an MC concentration of 100 μg MC/L in sterile controls, the reductions in the plant’s vigor were 29.59% GNPo; 36.78% LNP; 19.73% MRL; 53.05% PNP; 35.56% RDW; 18.84% SDW; and 36.54% SL. Concurrently, with the same concentration (100 μgMC/L MC), the tri-bacterial consortium A × B × C treatment, compared to the sterile controls with 0 μg MC/L, imparted an improvement of 25.74% GNPo; 4.89% LNP; 6.55% MRL; 52.58% PNP; 16.74% RDW; 34.22% SDW; and 26.17% SL.

### 3.5. Leaf Antioxidant Activities

The antioxidant activities in plant leaves irrigated with MC-contaminated water and the addition of toxin-tolerant bacteria were examined (Table S2). The table also includes statistical analyses of the Kruskal–Wallis test and the corresponding post hoc group comparison tests. All of the tested antioxidant enzymes showed similar directionality. Compared to toxin-free controls, treatments with 50 and 100 μg MC/L caused significant (*p* < 0.05) increases in antioxidant activities. On the other hand, bacterial additions reduced the level of antioxidant activities. The more stressed the plant is, the higher the level of antioxidant activity.

As the number of bacterial species in the consortium increased, the plant was less stressed, and the level of antioxidant activity decreased. The highest increases in antioxidant activity were observed in sterile controls with the highest toxin concentration, i.e., 100 μgMC/L. Compared to sterile controls and toxin-free treatments, the sterile control spiked with 100 μg MC/L showed the highest increase in activity of 103.4% CAT, 179.61% GST, 363.29% PPO, 294.97% POD, and 184.95% SOD. However, with the triple bacterial addition A × B × C, at 100 μg MC/L, the stress was contained, and the enzyme activities were reduced by 10.07% CAT, increased by only 2.02% GST, increased by only 56.96% PPO, reduced by 68.34% POD, and increased by only 12.47% SOD. Surprisingly, at 50 μg MC/L, in the sterile control, all enzymes increased in activity, ranging from 41.38% to 131.66%. In contrast, with the triple addition, at the same concentration, there was a complete suppression of stress via a reduction in antioxidant activity from 10.13 to 51.05%.

### 3.6. Plant Cell Structural Integrity Assessment 

The cellular integrity was assessed using Electrolyte Leakage (EL) and Lipid Peroxidation (MDA). The details for these parameters are shown in Table S3. MC concentrations led to highly significant cell damage in the plant for EL and MDA. For Electrolyte leakage, the greatest damage was found in the control without bacteria at 100 μg MC/L, where electrolyte leakage increased to 62.01%. However, triple bacterial consortia with 50 and 100 μg MC/L EL reduced to 1.25% or increased to 6.57%, respectively. Similarly, the most severe damage for MDA was 137.11% in the sterile control at 100 μg MC/L. The addition of the bacterial consortium A × B × C reduced MDA by 62.07, 59.13, and 32.71% for 0, 50, and 100 μg MC/L, respectively.

As a means of non-enzymatic plant defense, the impact of MC on and the role of PGPB in polyphenol (GAE) and ascorbic acid (AsA) contents have been provided in Table S3. Polyphenols increased significantly (*p* < 0.001) with increasing MC concentrations and decreased considerably with the complexity of the bacterial consortia. While, at 100 μg MC/L, the sterile control recorded an increase of 124.9% in GAE, the bacterial consortium A × B × C recorded a reduction of 49.32, 42.63, and 18.88% for 0, 50, and 100 μg MC/L, respectively. For ascorbic acid, we noted that, as MC increased, the AsA level increased as well. Additionally, as the number of bacterial strains increased, the AsA level also increased. Therefore, at 100 μg MC/L, in the triple bacterial consortium, the AsA increased by 154.71% as compared to the sterile control at 0 μg MC/L.

### 3.7. Nutritional Value

#### 3.7.1. Mineral and Phosphorus Richness of the Fruits

The mineral contents of Calcium, Potassium, and Sodium in the fruits differed significantly (*p* < 0.001) between the treatments without and with MC. The Ca and K contents decreased as the MC concentrations increased, whereas the Na content increased as the MC concentrations increased. In contrast, the Ca and K contents improved with bacterial addition, while Na contents decreased (Table S4). The phosphorus contents in both leaves and fruits were significantly reduced at increasing MC concentrations, *p* < 0.001. The highest reduction reached 31.22% in fruits, while in leaves, this reduction reached 55.52% at 100 MC μg/L in sterile control. Bacterial additions significantly improved the phosphorus content of the leaves and fruits, *p* < 0.001. Following the triple bacterial consortium, at 100 μg MC/L, the P content increased by 7.86% compared to a reduction of 31.22% in the sterile control. However, in leaves, the triple bacterial consortium caused a decline of 1.86% vs. a decrease of 55.52% in the sterile control.

#### 3.7.2. Protein and Sugar Content of Fruits and Leaves

The protein and sugar patterns in the leaves and fruits changed significantly under MC stress and bacterial addition (Table S5). Increasing MC concentrations resulted in a highly significant protein decrease for both soluble and crude proteins in leaves and fruits (*p* < 0.001). Conversely, bacterial addition improved the protein content significantly (*p* < 0.001). On the other hand, MC-caused decreases caused by MC appeared more severe in leaves than in fruits. Conversely, the increase in protein due to bacterial additions was higher in fruits than in leaves.

The sugar trend in leaves and fruits diverged in the presence of MC. Sugar concentration decreased in the fruits with increasing MC concentrations, whereas, in leaves, the sugar contents increased with increasing MC concentrations. However, with bacterial addition, the amount of sugar in the fruits increased significantly (*p* < 0.001), while the sugar in the leaves decreased significantly (*p* < 0.01). 

### 3.8. Principal Component (PC) Analyses

To warrant the satisfaction of the conditions for principal component analysis, the determinant of the correlation matrix was low but not 0, i.e., 4.74 × 10^−34^, while the Kaiser–Meyer–Olkin (KMO) adequacy index was 0.962, which corresponds to the so-called superb adequacy level (KMO > 0.9). As for the inter-variable correlation shown by Bartlett’s Sphericity Test, the statistic was highly significant, with H (465) = 6066.723, *p* < 0.0001.

Assuming that all variables contributed equally to the variation of the principal components (3.23%), only two principal components (PC) contained more information represented by a single variable. These are PC1 to PC2, with a cumulative variation of 85.78%. The contribution to this variance was 80.86% and 4.92% for PC1 and PC2, respectively.

The total squares of all loadings (lo) for an individual principal component must equal one. In this study, if all the variables contributed equally to the variation of the main PC, the loading for each of the 31 variables would be 0.179. Thus, a loading greater than 0.179 was considered significant, and their corresponding variables were retained as contributing more to the PC.

**Figure 7:**
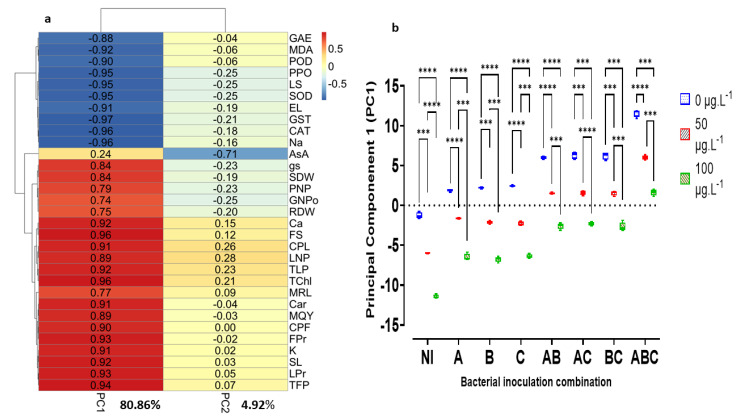
Heatmap (**a**) of the first two principal components, PC1 and PC2, capturing 85.78% of the variation expressed by the 31 variables studied. The values inside the map correspond to the positive and negative correlations of the different variables with respect to the Principal Components 1 and 2. Variables are defined as: stem length (SL), leaves number per plant (LNP), main root length (MRL), pod number per plant (PNP), grain number per pod (GNPo), shoot dry weight (SDW), root dry weight, stomatal conductance (gs), maximum quantum yield (MQY), total chlorophyll (TChl), carotenoids (Car), ascorbic acid (AsA), total fruit phosphorus content (TFP), total leaf phosphorus content (TLP), catalases (CAT), glutathione transferases (GST), polyphenol oxidase (PPO), peroxidase (POD) polyphenols (GAE), electrolyte leakage (EL), malondialdehyde (MDA), leaf soluble sugars (LS), fruit soluble sugars (FS), leaf soluble proteins (LPr), superoxide dismutase (SOD), calcium content in fruits (Ca), potassium content in fruits (K), sodium content in fruits (Na), crude protein content in leaves (CPL), and crude protein content in fruits (CPL); and general visualization (**b**) of the negative impact of MC and the overall gain promoted by bacterial addition on the overall pea condition by projecting MC contaminations and bacterial additions onto the first principal component. The stars correspond to statistical significance at different levels as ns= not significant; *** = *p* < 0.001; **** = *p* < 0.0001.

Figure 7 is a heatmap showing the contribution of each variable to the variation of the two principal components. Two clusters stand out in the figure. Conversely, the maximum variation is almost represented on PC1 with 80.86%, while PC2 captures only 4.92% of the variation. While only four variables—RDW, SDW, PNP, and MRL—have an absolute value of loading lower than the average contribution of a single variable of 0.179, all other variables, except AsA, that contribute strongly to the variability of PC2 with a lot of −0.649, have loadings higher than 0.179, indicating their importance in the representativeness of the variation of PC1.

On the other hand, the parameters related to stress or indicating the damage caused to the plants correlate strongly in a negative way to the PC1. In contrast, all the other variables representing the physiological and nutritional state of the plant are positively correlated with PC1.

The two principal components of the two newly created super-variables were used to understand the impact of MC on the plant and the role of bacterial addition in buffering this stressful situation created by the toxin. The results of the Kruskal–Wallis analysis of PC1 vs. the bacterial addition and MC concentration are highly significant: H(7) = 29.133, *p* < 0.0001 and H(2) = 59.504, *p* < 0.0001 for bacterial addition and MC concentration, respectively. The post hoc test shows highly significant differences for the tri-bacteria consortium A × B × C at the 5% cutoff level. On the other hand, 50 and 100 μg MC/L treatments significantly differed from each other and the sterile control. Figure 7 shows the projection of the two independent variables studied: the bacterial addition and the MC concentration on PC1.

## 4. Discussion

### 4.1. Plant Growth-Promoting Properties of Planktonic and Rhizosphere Bacteria (PGPB)

Bacterial plant growth promotion by bacteria is a hot research topic that interests many researchers searching for the most economical way to increase yields naturally. Thus, PGPB are used as an alternative to conventional artificial fertilizers to maximize crop yields [68,69,70,71] and protect the environment [39]. Typically, these bacteria are isolated in the plant’s rhizosphere [36,68,72], but they can also emanate from water [73]. The common characteristics of these bacteria are their abilities to colonize the plant’s rhizo-phyllosphere and establish a mutual relationship with the host. PGPB enhance plant growth by facilitating mineral uptake, phosphorus and potassium solubilization, and the production of plant growth molecules such as phytohormones (Auxin and Gibberellic acid) [70,74], exopolysaccharide production, and the fixation of atmospheric nitrogen [75,76]. The most common PGPG genera are *Rhizobium*, *Bacillus*, *Acinetobacter*, *Burkholdera*, *Promicromonospora*, *Enterobacter*, *Pseudomonas*, *Streptomyces*, *Nocardiopsis*, *Azospirillum*, *Klebsiella*, *Alcaligen*, *Serratia*, *Xanthomonas*, and *Brevundimonas* [33,34,71,77,78].

In this study, the 16S RNA-characterized *Brevindimonas* sp strain showed PGPB properties of auxin production with 46.19 ± 1.13 μg IAA/mL and 92.66 ± 8.36 μg CR/OD 600 for Exopolysaccharides (EPS) and 0.32 ± 0.0008 mg/L of phosphorus solubilized in a liquid NYRIB tricalcium phosphate medium. However, the strain did not show a phosphorous solubilization capability on the NYRIB solid medium. Our results agree with those of Kumar & Gera (2014) [79] and Rana et al. (2011) [80]. While Kumar & Gera [79] found that *Brevundimonas* sp. could produce up to 364.1 ± 0.750 µg IAA/mL and 0.954 ± 0.006 ug NH_4_^+^/mL, the strain was not able to solubilize phosphorus or produce any siderophore. However, the strain carried the *nif*H gene and showed nitrogen fixation by the acetylene reduction assay. Similarly, Rana et al. (2011) [80] reported that, between two strains (AW7 and AW9) characterized by a 99% similarity to *Brevundimonas* sp., only AW7 could produce NH4+ and solubilize phosphorus. Yet, AW7 and AW9 produced 13.96 ± 2.0 ug IAA/mL and 12.96 ± 1.8 ug IAA/mL, respectively. Though rarely used as PGPB bacteria, the *Starkeya* sp. strains in this study performed well in EPS and IAA production and the solubilization of phosphorus and potassium SLB1 > SLB3. Similarly, Agafonova et al. (2017) [81] described a *Starkeya novella* strain with phosphorus-solubilizing and IAA-producing capabilities, which also exhibited the *nif*H gene for nitrogen fixation. Zakhia et al. (2006) [82] isolated *Starkeya* strains whose *nif*H sequences were closely related to *Sinorhizobium meliloti,* indicating the general potential of *Starkeya* strains for nitrogen fixation.

For the first time, Lin et al. (2014) [83] showed that *Brevundimonas* sp. could be used to control the proliferation of *Synechococcus* sp. BN60 blooms via a 48.6% reduction of cyanobacterial biomass in 6 days, killing up to 91.8% of *Microcystis aeruginosa* 9110. Beyond the inhibitory activity of *Brevundimonas* sp. on toxic cyanobacteria, Massey & Yang (2020) [84] reported that bacteria isolated from the mucilage of *M. aeruginosa* colonies during a mass bloom in a French pond were able to fully degrade MC-LR and Des-MCLR. In this study, the strains SLB1 and SLB3, both *Starkeya* sp., revealed a higher tolerance to MC than the *Brevundimonas* strain (RTC10). While no studies were found linking *Starkeya* sp. to MC degradation, the bacteria of the *Starkeya* genus seem to be involved in the depollution and degradation of recalcitrant molecules. When supplied with carbamazepine as a sole carbon source, the strains of *Starkeya* sp. C11 and *Rhizobium* sp. C12 were able to degrade 30% of 10 mg carbamazepine/L. Sun et al. (2019) [85] described a metabolic pathway of *Starkeya* strains by which carbamate pesticides are degraded, which are esters of carbamic acid toxic to vertebrates and invertebrates because they inhibit the acetylcholinesterase (AChE) activity. In addition, the ability of *Starkeya* strains to destroy difficult-to-manage organic pollutants has been demonstrated, i.e., Naphthalene [86] and Polychlorinated biphenyls (PCBs) [87].

### 4.2. Plant Physiological Response to MC Stress and Bacterial Interactions

MC can cause plant growth retardation in many ways [88]. Photosynthesis, the main characteristic of plants, is the first characteristic to be affected by MC-induced stress due to reactive oxygen species (ROS) produced in the stressed plant [16]. In this study, 100 μg MC/L resulted in a decrease of 77.28% in total chlorophyll (a + b), a 41.73% decrease in stomatal conductance, and a 10.45% decrease in the maximum quantum yield (Fv/Fm) [89]. Saqrane et al. (2009) [19] observed a 70% decrease in Fv/Fm in *Pisum sativum* following extract 100 μg MC/Lt exposure. Additionally, Lahrouni et al. (2013) [90] recorded a significant reduction in Fv/Fm of up to 15% in *Vicia faba* L following plant exposure to 100 μg MC/L. 

Here, 100 μg MC/L reduced carotenoids up to 61.64% compared to the toxin-free control. Regarding the effect of MC on the chlorophyll content in *Pisum sativum*, however, our results diverge from those of Saqrane et al. (2009) [19]. The authors found no significant differences in the Chl (a + b) contents in *Pisum sativum* and *Triticum durum* exposed to 2.1 and 4.2 mg MC/L, i.e., concentrations that are 42 times higher than those used in this study. However, they recorded a 22–25% decrease in Chl (a + b) in *Zea mays* and *Lens esculenta* at 2.1 and 4.2 mg MC/L. The lack of difference at 2.1 and 4.2 mg MC/L MCs may be due to the extract composition in terms of MC variants. In fact, the extract used was composed of six MC variants, with MC-LR representing only 27%. In contrast, we used an extract that contained almost only the toxic MC-LR variant, comparable to the commercial standard [29]. The reduction of a plant’s photosynthetic capacity may be due to gas exchange impairment related to reduced stomatal conductance (gs) due to high functional disturbance caused by stress [91,92]. Indeed, under stress, stomata are closed [93], and photosynthesis is greatly reduced [94]

Inoculating a plant with PGPB bacteria improves the plant physiology under stress conditions [36,39,95]. While increasing MC concentrations caused significant reductions in TChl, MQY, gs, and Car, the presence of bacteria improved the plant’s physiology compared to the sterile control. In addition, the physiological state improved with the added number of bacterial strains. The plant inoculated with just a single strain performs better than the sterile control but when the inoculation involves two strains or three strains the plant perormace is even better. Overall, the triple bacterial consortium confers significant improvements compared to the sterile plant [29].

The MC-tolerant *Ensifer meliloti* strain enabled a significant increase in the pigments of *Medicago sativa* compared to the non-MC-tolerant strain [29]. Similarly, Lahrouni et al. (2013) [90] showed that chronic exposure to MC reduced the pigment content in *Vicia faba.*, even when plants were inoculated with MC-tolerant Rhizobium strains. However, the authors recorded significant differences when inoculating with an MC-tolerant *Rhizobium* strain and a non-tolerant strain. 

### 4.3. Morphometric and Yield Indicators

The plant’s physiological disorders caused by MC affect growth and lower yields [24,88,96]. However, PGPB bacteria can impart significant growth advantages to plants even under extreme oxidative stress conditions such as those caused by MC [29,40,41,43]. The extent of damage caused by MC depends on the dose and the type of plant [19]. In our study, the chronic exposure of the sterile pea to the highest concentration of 100 μg MC/L caused a reduction of 18.84%, 19.73%, 29.59%, 35.56%, 36.54%, 36.78%, and 53.05% for SDW, MRL, GNPo, RDW, SL, LNP, and PNP, respectively. Bacterial addition increased the vigor in all these parameters. For the same concentration, the triple addition of our three bacterial strains permitted significant increases of 34.22%, 6.55%, 25.74%, 16.74%, 26.17%, 4.86%, and 52.58% for SDW, MRL, GNPo, RDW, SL, LNP, and PNP respectively. Our results are consistent with those of Zhu et al. (2018) [97]. They noted that 10 μg MC/L decreased the plant height, stem diameter, leaf area, and root dry weight of cucumber by 70% at the seedling stage, 84% at the early flowering stage, and about 90% at the fruiting stage compared to the toxin-free control. The authors also reported that the degrees of deterioration increased with concentrations between 100 and 1000 μg MC/L. Petrou et al. (2020) [96] stated that environmental MC doses supplied via irrigation did not have a negative effect on the nitrifying rhizosphere microbiome but affected radish leaf fresh weight and taproot. Similarly, Lahrouni et al. (2016) [43] demonstrated the importance of inoculation with a nitrogen-fixing Rhizobium PGPB to protect the plant against MC toxicity. Moreover, the authors showed that plants fed with NH_4_NO_3_ nitrogen and watered with 100 μg MC/L recorded a decrease in shoot weight by 27% and a decrease in root weight by 37% compared to 17% and 30% in plants inoculated with the N_2_-fixing Rhizobium strain, respectively. The bacteria used in this study showed several plant growth-promoting aspects.

MC degradation abilities were not tested in this study. However, we proved that the isolates could grow on a mineral salt medium dosed with 1000 μg MC/L as the only carbon and nitrogen source for bacteria.

Growth and yield advantages, such as the number of pods per plant and the number of seeds per pod, increased to 134.74 and 88.76%, respectively, when adding a consortium of all three bacteria without toxins and to 52.58 and 25.74% at 100 μg MC/L, respectively. This suggests significant plant growth promotion in the presence of bacteria, even when MC concentrations are high. The PGPB properties of these bacteria include the secretion of phytohormones such as auxin, gibberellic acid, and the solubilization of phosphorus, potassium, and exopolysaccharides production, and siderophores allow for significantly accelerated plant growth compared to sterile control treatments [39,71,75,98,99]. On the other hand, the PGPB properties control the translocation of toxic elements to the plant, thereby reducing the generation of reactive oxygen species (ROS) and consequently decreasing oxidative stress [36]. Nevertheless, we speculate that the accelerated plant growth facilitated by the various properties of PGPB buffers the toxic effects that a stunted plant would experience under biotic or abiotic stress.

### 4.4. Plant Antioxidant Response and Cellular Integrity

Reactive oxygen species (ROS) are products generated in a normal plant development cycle as by-products of essential mechanisms such as photosynthesis and respiration [100,101]. Furthermore, ROS are indispensable in several fundamental biological processes, including cellular proliferation and differentiation [102]. However, although a certain level of ROS is necessary to support essential cellular functions and viability, ROS accumulate in a cell under biotic or abiotic stress beyond the capacity of the enzymatic and non-enzymatic antioxidant systems inducing cell toxicity and death [103,104,105]. 

The optimal functioning of an organism depends on cellular integrity. Malondialdehyde (MDA) and electrolyte leakage (EL) are two critical stress indicators [25,77,106]. During stress, MDA and EL increase dramatically, leading to irreversible damage to the plasma membrane, chloroplast, and mitochondria, disrupting cell organelles’ stability and selective permeability [107]. Here, we demonstrate that increasing concentrations of MC significantly increase MDA and EL levels, while the presence of bacteria decreases these levels. The highest reduction appears progressively with an increasing number of bacteria added. The ROS generated during stress are the basis for cell damage via the peroxidation of the cell membrane and mitochondrial and chloroplast lipids, increasing MDA and EL [108].

Similarly, several authors have reported that the exposure of crop plants to MC at concentrations as low as 5 μg MC/L resulted in a significant increase in MDA [23,25,106,109]. During oxidative stress, lipid peroxidation changes the cell membrane, augmenting EL due to osmotic imbalance within the cell [56,110]. Similar conclusions have been obtained by Kang et al. (2014) [36] and Batool et al. (2020) [96]. Our results show that plants inoculated with PGPB exhibit low EL values and MDA values under MC oxidative stress conditions.

Furthermore, we show that increasing MC concentrations exert a stress-elevating effect on the contents of all antioxidant enzymes tested. At the same time, bacterial addition significantly decreases the levels of these antioxidant enzymes. The high concentration of antioxidant enzymes suggests an equally high concentration of ROS and vice versa. For enzymatic defenses, we noted a similar pattern. On the one hand, in the sterile control, at 100 μg MC/L, the rates of increase reached 100.3%, 179.61%, 363.29%, 294.97%, and 184.95% for CAT, GST, PPO, POX, and SOD, respectively. On the other hand, we observed a progressive decrease in these rates as the number of added bacteria increased. At 100 μg MC/L, the lowest levels are found in the consortium with all three bacterial strains. Therefore, our results suggest that 100 μg MC/L caused the highest level of oxidation in the plant and hence the intense recruitment of antioxidant enzymes to counteract the challenge. However, bacterial presence softens the ROS damage. 

Stored beyond the required levels and cellular elimination capacities, ROS trigger cellular apoptosis by disrupting intracellular redox homeostasis and causing irreversible oxidative modifications of lipids, proteins, or DNA, which can activate oxidative stress-induced apoptotic signaling [111,112]. Drobac et al. (2017) [23], investigating the toxic effects of MC on the antioxidant capacity of *Capsicum annuum* leaves and fruits, reported that the concentration of 2.22–100 µg/mL of MC increased the activities of antioxidant enzymes and caused the accumulation of polyphenols and Malondialdehyde, suggesting that the exposure to MC reduced the antioxidant capacity of the experimental plants and altered the homeostasis of *C. annuum*. Here, increasing concentrations of MC to 50 and 100 μg MC/L induced an increase of 38.29–124.9% in the sterile treatments compared to the MC-free control. On the other hand, bacterial presence decreased MC stress in *Vicia faba* [29]. The polyphenol levels decreased as the number of bacterial strains involved in the addition increased (Table S3).

Furthermore, Kang et al. (2014) [36] showed that PGPB improved plant conditions under salt stress by reducing catalase, peroxidase, polyphenol oxidase, and total polyphenol compared to the sterile control. Indeed, the role of polyphenols and flavonoids is essential, as they can counteract ROS generation by inhibiting enzymes and chelating trace elements involved in the free radical production, trapping ROS and up-regulating or shielding antioxidant defenses [23,113].

Another important result of this study concerns the ascorbic acid (AsA) content. Like polyphenols and carotenoids, AsA is a potent non-enzymatic antioxidant with multiple roles [105,113,114]. AsA can scavenge and neutralize excess ROS resulting from stress, such as superoxide radical anion (O_2_**^−^**), hydrogen peroxide (H_2_O_2_), hydroxyl radicals (OH·), singlet oxygen (1O_2_), and reactive nitrogen species (RNS) [101,115,116]. In this study, we observed that the level of AsA increased significantly with increasing MC concentrations. Intriguingly, the AsA levels also increased with the complexity of strains in the bacterial addition (Table S3). This result contradicts the findings of Machado et al. (2017) [31], who reported a significant decrease in the AsA content in roots of carrots irrigated with MC-contaminated water with 10 and 50 μg MC/L. AsA can stimulate DNA synthesis in root meristems by shortening the G1 phase and stimulating entry into the S phase, thereby allowing for cell proliferation and elongation [117,118]. In addition to its ability to control cell division, elongation, and differentiation, vitamin C also manages programmed cell death upon severe stress [116,119]. Our results are in agreement with the findings of Abdelaal et al. (2021) [108] and Patel et al. (2020) [120] regarding the AsA trend in a plant inoculated with PGPB bacteria and subjected to stress. The authors report that PGPB bacteria significantly increase the level of AsA compared to sterile controls. Here, we noted that, at 100 μg MC/L, the ascorbic acid contents increase considerably, with the number of added bacterial strains varying from ca. 65%, ca. 83%, and ca. 154% for mono-, bi-, and tri-bacterial consortia. Given the multifunction of this vitamin in the metabolism and physiology of the plant, apart from the fact that PGPB bacteria result in an increase in vitamin C as a nutritional reserve in the fruit, we can postulate that, as a response to MC-induced oxidative stress, PGPB bacteria (a) stimulate the increase of AsA to counterbalance the damage caused by ROS to the plant or (b) initiate programmed cell death when the plant’s reparative capacities have been exceeded

### 4.5. Plant Nutritional Value

Consumers choose food depending on, among other things, the organoleptic properties and the importance of nutrient properties necessary for good health [121,122]. Peas’ main nutrient values include carbohydrate components, proteins, fibers, vitamins, minerals, and other phytochemicals [121,123]. Here, increasing MC concentrations significantly lowered the soluble and crude protein contents, the phosphorus in fruits and leaves, and the calcium, potassium, and sugar in fruit. However, increasing MC concentrations enhanced the sodium content in the fruits and the sugar in the leaves. Simultaneously, bacterial addition exerted a corrective action opposite to that of MC. Cao et al. (2018) [25] reported that 50 and 500 μg MC/L addition to *Oryza sativa* significantly increased dissolved organic carbon (DOC) and carbohydrates in the root. They hypothesized that the variability of minerals and other organic nutrients could be due to cell membrane imbalance via ROS, leading to lipid peroxidation and EL. Further studies confirmed that plant exposure to MC leads to significant variations in Ca, Na, K, and P minerals depending on the plant organ [17,19,90]. While these minerals increase in roots [17,19], they decrease in leaves and fruits [27,124]. 

Studies on the protein content in leaves or fruits when plants are irrigated with MC-contaminated water are contradictory. While Liang et al. (2021) [124] observed an increase in the soluble protein in rice fruits after 10 days of irrigating plants with 10 μg MC/L, El Khalloufi et al. (2011, 2012) [17,42] also reported an increase in soluble protein in the roots and leaves of *Lycopersicon esculentum* and *Medicago sativa* after 30 days of irrigation with 22,240 μg MC/L. However, Haida et al. (2022) [109] recorded a decrease in the total protein in strawberry leaves and roots after 60 days of irrigation with 20 μg/L. In a study by Bittencourt-Oliveira et al. (2016) [20], the total protein in lettuce leaves increased when irrigated for 15 days with MC ranging from 0.65–6.5 μg MC/L. However, the protein contents dropped significantly at 13 μg MC/L. Similary, Liang et al. (2021) [124] and Zhu et al. (2018) [97] demonstrated, in two different model plants, i.e., rice and cucumbers, that concentrations <100 μg MC/L boosted the total protein contents, while concentrations between 100–1000 μg MC/L significantly decreased plant protein contents. The increase in proteins may be a defense reaction of the plant to ROS excess to protect itself from oxidation by mobilizing its antioxidant resources.

More interestingly, with MC-induced ROS stress, the sugar content in leaves increases, while it decreases in fruits. Haida et al. (2022) [109] observed a significant increase in the total sugars in strawberry roots and leaves after 60 days of exposure to 20 μg MC/L. However, Liang et al. (2021) [124] and Zhu et al. (2018) [97] argued that MC concentrations as low as 10 μg MC/L promote sugar content in cucumbers and rice. The authors also reported that MC concentrations ranging from 100–1000 μg MC/L decreased the total sugar contents in these fruits. In our study, we observed an increase in total sugar in leaves and a decrease in total sugar in fruits at concentrations of 50 and 100 μg MC/L. However, applying PGPB bacteria decreased the sugars in the leaves and increased them in the fruits. 

The decrease in sugar in fruits could be related to the photosynthetic capacities dampened by MC via the disruption of stomatal activity through reduced transpiration. The inhibition of the photosynthetic mechanism could then lessen the carbohydrate packing in fruits. On the other hand, the increase in sugars in roots and leaves may be a response of the plant to regulate the osmolarity disturbed by ionic imbalance and EL [93,95].

This study is the first to clearly demonstrate the role of PGPB bacteria in significantly increasing the nutritional quality of plants, especially in sugars, proteins, and minerals, when these plants are irrigated with MC concentrations up to 100 μg MC/L. Recent work by Lahrouni et al. (2016) [43] showed that microcystin-tolerant Rhizobium protects faba bean plants and improves nitrogen assimilation when grown in the presence of 100 μg MC/L.

Finally, the dimensional reduction (Figure 7a) of the studied problem reveals that EL, MDA, LS, Na, and antioxidant enzymes (CAT, GST, PPO, POD, and SOD) were negatively correlated by more than 90% with the main component 1, while physio-morphological indicators—TChl, carotenoids, and stem length—and nutritional characteristics were positively correlated with this same component by more than 90%. Thus, the projection of the new coordinates of the principal component 1 on the orthogonal axes (Figure 7b) clearly shows that MC negatively affect all parameters represented by the dimension. Bacterial addition results in beneficial interactions that allow the plant to maintain optimal functioning and increase its growth and production potential. We observed that the number of added bacterial strains increases the beneficial effect of the consortium on the plant.

In this study, we did not test the ability of PGPB to degrade the MC into by-products that are not harmful to the plant. Therefore, despite the growth enhancement and other bacterial benefits to the plant, MC translocation and potential accumulation in the plant organs inoculated with PGPB should be investigated.

## 5. Conclusions

Crop irrigation with MC-contaminated water leads to systemic disorders in the plant, both on the physiological and biochemical levels. Increasing concentrations of MC significantly reduce pea growth and grain yield. However, PGPB improved plant growth and increased biomass and grain yield even in the presence of MC. Morphological changes perceptible to the naked eye, such as the stem height or leaf number increasing after bacterial addition, result from cascades of induced enzymatic reactions. MC generated ROS that increased EL and MDA contents and activated various antioxidant enzymes involved in ROS scavenging ROS to heal the distressed plant. While MC elevated the MDA and EL contents as well as the antioxidant enzymes, including SOD, CAT, PPO, POD, and GST, indicative of chronic stress, bacterial presence significantly reduced the concentration of all these parameters, suggesting that PGPB mitigates or suppresses MC-induced oxidative stress. Moreover, MC impaired pea nutritional quality by reducing the mineral contents and decreasing the total sugars and proteins in fruits and leaves. PGPB consortia improved the overall pea nutritional value. In short, this study is the first to isolate and characterize the PGPB activity of bacterioplankton associated with toxic cyanobacterial blooms and use them to promote plant growth under MC stress and to improve the nutritional value of fruit. However, further investigations on the fate of MC in the rhizosphere and the potential accumulation of these toxins in fruit are required to confirm the broad application of PGPB in increasing crop yields in modern agriculture.

## Figures and Tables

**Table 1:** Phosphorus and potassium solubilization in liquid and solid media and other PGR parameters: exopolysaccharide production and indole acetic acid by bacterial strains.

Isolates	Exopolysaccharides μg of CR/OD 600	K Solubilization on Solid Media DHk/DCk	Indole Acetic Acid (μg/mL)	P Solubilization on Solid Media DHp/DCp	Soluble Phosphorus (mg/L)
**SLB1**	231.07 ± 26.22 a	2.12 ± 0.75 a	231.5 ± 2.16 a	1.84 ± 0.4 a	0.27 ± 0.0023 a
**SLB3**	120.17 ± 27.76 b	1.00 ± b	144.0 ± 2.41 b	1.00 ± b	0.29 ± 0.0004 b
**RTC10**	92.66 ± 8.36 b	0.00 ± c	46.19 ± 1.13 c	0.00 ± c	0.32 ± 0.0008 c

Values are means of three replicates. Differences in the data were considered significantly different at the probability level of *p* ≤ 0.05 (indicated by different letters). Means not sharing the same letter are significantly different.

## Data Availability

Not applicable.

## Supplementary Materials

The following supporting information can be downloaded at: https://www.mdpi.com/article/10.3390/microorganisms10081511/s1, Figure S1: Growth kinetics by optical density at 600 nm of bacterial isolates on minimal salt media (MSM) spiked with 1000 µg MC/L extract. SLB1 and BL10 are the strains isolated from *Microcystis*-bloom, B = SLB3 is the strain isolated from MC-contaminated water, and C = RTC10 is the strain isolated from agricultural MC-contaminated soil; NI corresponds to the sterile control without bacteria; Table S1: Physicochemical properties of soil used in pots and irrigation water. Means not sharing the same letter are significantly different. Table S2: Effects of bacterial inoculation and increasing concentrations of Microcystins on antioxidant enzymes: Catalases (CAT), Glutathione transferases (GST), Polyphenol oxidase (PPO), Peroxidase (POD), Superoxide dismutase (SOD) in peas, *Pisum sativum*. Table S3: Effects of bacterial inoculation and increasing concentrations of Microcystins on Electrolyte leakage (EL), Malondialdehyde (MDA), ascorbic acid (AsA), and Polyphenols (GAE) in peas. Table S4: Effects of bacterial inoculation, increasing concentrations of Microcystins on total leaf phosphorus (TLP), total fruit phosphorus (TFP), and fruit minerals: Na, Ca, and K in peas. Table S5: Effects of bacterial inoculation and increasing concentrations of Microcystins on sugars and proteins in both the leaves and fruits in peas.
