# Supplementary material for: Bacterioplankton Associated with Toxic Cyanobacteria Promote Pisum sativum (Pea) Growth and Nutritional Value through Positive Interactions
Source: Microorganisms. 2022 Jul 26;10(8):1511. doi: 10.3390/microorganisms10081511 (PMC9394358; doi:10.3390/microorganisms10081511)
Supplement: Supplementary file 1 [file microorganisms-10-01511-s001.zip › microorganisms-1826559-supplementary.pdf]

Supplemental Data

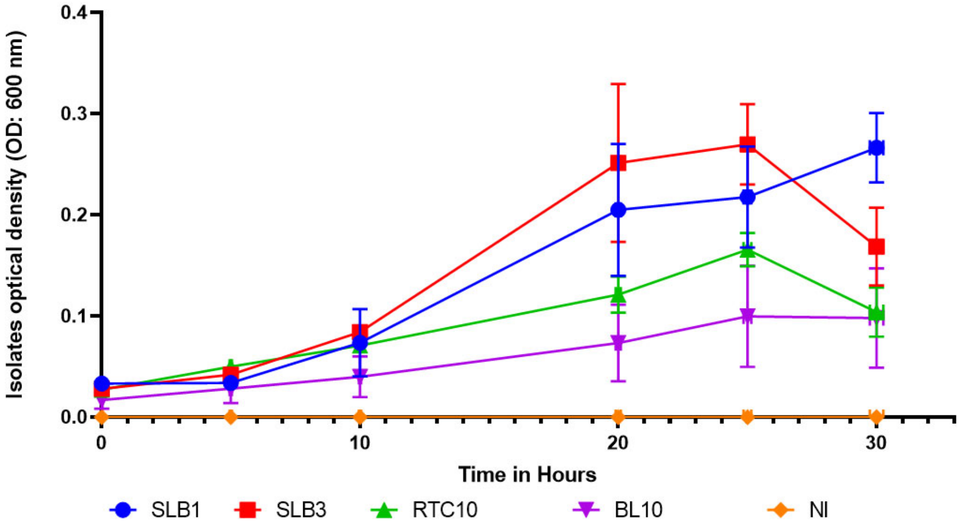

.

Figure S1: Growth kinetics by optical density at 600 nm of bacterial isolates on minimal salt media (MSM) spiked with 1,000 µg MC/ L extract. SLB1 and BL10 are the strains isolated from *Microcystis*-bloom, SLB3 is the strain isolated from MC-contaminated water, and RTC10 is the strain isolated from agricultural MC-contaminated soil; NI corresponds to the sterile control without bacteria

Table S1: Physicochemical properties of soil and irrigation water.

substrate	%TKN	%N-NH4	N-NO3	% TOC	% CaCO3	EC µs.cm-1	pH	Assimilable phosphorous (PO_4^{3-})
**Soil**	0.11±0.005	0.009±0.001	40±0.01mg.100 g-1	6.79±1.63	7.44±0.55	787.00±1.00	8.22±0.16	639.98±0.002ug.g-1
**water**	-	-	33.06±4.104 mg.L-1	-	-	1718±27	6.98±0.087	20.43±3.1ug.L-1

Values are means of three replicates. Means not sharing the same letter are significantly different. TKN stands for Total Kjeldahl nitrogen OC stands for total organic carbon, and EC stands for electrical conductivity

Table S2: Effects of bacterial inoculation and increasing concentrations of Microcystins on antioxidant enzymes: Catalases (CAT), Glutathione transferases (GST), Polyphenol oxidase (PPO), Peroxidase (POD), Superoxide dismutase (SOD) in pea, *Pisum sativum*

Bacterial Inoculations	MC treatment (µg.L-1)	Catalases U. mg-1 protein. g-1 FW	% Increase (+) or Decrease (-)	glutathione transferases U. mg-1 protein. g-1 FW	% Increase (+) or Decrease (-)	Polyphenol oxidase µmol of catechol.mg-1protein.min-1	% Increase (+) or Decrease (-)	Peroxidase µmole of oxidized guaiacol.mg-1protein.min-1	% Increase (+) or Decrease (-)	Superoxide dismutase U. mg-1 protein. g-1 FW	% Increase (+) or Decrease (-)
**NI**	0	37.65±1.25g		24.72±3.15gh		0.79±0.02jk		1.99±0.29f		223.26±16.44h	
	50	53.23±1.01d	41.38	42.44±4.26d	71.68	1.58±0.07d	100	4.61±0.11c	131.66	370.54±7.70d	65.97
	100	76.58±1.98a	103.4	69.12±1.39a	179.61	3.66±0.07a	363.29	7.86±0.66a	294.97	636.18±12a	184.95
**A**	0	27.66±1.83i	-26.53	18.31±0.8ij	-25.93	0.9±0.02hi	13.92	2.08±0.1f	4.52	198.41±1.53i	-11.13
	50	41.04±5.39f	9	33.91±2.42e	37.18	1.4±0.04e	77.22	3.23±0.15d	62.31	294.83±20.26e	32.06
	100	59.19±2.01c	57.21	59.75±1.49b	141.71	2.04±0.08bc	158.23	5.19±0.4b	160.8	487.73±35.27b	118.46
**B**	0	17.9±2.22k	-52.46	15.11±0.77jk	-38.88	0.92±0.13gh	16.46	2.08±0.13f	4.52	199.12±7.13hi	-10.81
	50	33.09±3.16h	-12.11	29.76±1.87f	20.39	1.4±0.06e	77.22	3.42±0.19d	71.86	297.12±13.53e	33.08
	100	57.17±2.81c	51.85	55.84±6.58c	125.89	2±0.1c	153.16	5.11±0.41b	156.78	485.04±18.03b	117.25
**C**	0	23.03±1.8j	-38.83	18.94±0.99i	-23.38	0.84±0.07ij	6.33	2.06±0.07f	3.52	197.32±7.52i	-11.62

	50	40.55±2.11fg	7.7	41.17±3.98d	66.55	1.37±0.05e	73.42	3.32±0.22d	66.83	284.33±11.16ef	27.35
	100	62.6±2.1b	66.27	60.56±2.66b	144.98	2.11±0.13b	167.09	5.24±0.25b	163.32	494.97±17.97b	121.7
**AB**	0	13.05±1.69l	-65.34	10.66±0.7l	-56.88	0.43±0.03lm	-45.57	1.38±0.13g	-30.65	180.92±9.1ij	-18.96
	50	28.27±1.48i	-24.91	26.1±2.71g	5.58	1±0.06g	26.58	2.65±0.1e	33.17	268.7±29.51fg	20.35
	100	49.24±1.18e	30.78	44.28±2.37d	79.13	1.6±0.05d	102.53	3.17±0.12d	59.3	408.26±13.1c	82.86
**AC**	0	9.81±1.87m	-73.94	5.16±0.87m	-79.13	0.38±0.04mn	-51.9	1.46±0.14g	-26.63	189.56±5.87ij	-15.09
	50	25.96±3.67ij	-31.05	21.76±0.88hi	-11.97	0.99±0.02g	25.32	2.51±0.13e	26.13	270.28±23.34fg	21.06
	100	39.22±2.66fg	4.17	35.68±1.66e	44.34	1.59±0.04d	101.27	3.27±0.12d	64.32	410.63±18.23c	83.92
**BC**	0	6.74±1.2mn	-82.1	10.53±2.16l	-57.4	0.5±0.09l	-36.71	1.43±0.05g	-28.14	172.95±13.6j	-22.53
	50	27.88±0.69i	-25.95	21.71±2.38hi	-12.18	0.93±0.02gh	17.72	2.43±0.15e	22.11	255.21±36.16g	14.31
	100	48.04±1.6e	27.6	43.12±2.98d	74.43	1.61±0.06d	103.8	3.19±0.09d	60.3	407.32±11.16c	82.44
**ABC**	0	4.45±0.19n	-88.18	3.91±0.57m	-84.18	0.3±0.02n	-62.03	0.85±0.01h	-57.29	111.61±1.49k	-50.01
	50	18.43±0.69k	-51.05	13.79±1.89kl	-44.22	0.71±0k	-10.13	1.24±0.02g	-37.69	167.52±7.6j	-24.97
	100	33.86±3.3h	-10.07	25.22±2.22gh	2.02	1.24±0f	56.96	0.63±0.04h	-68.34	251.1±13.06g	12.47
**Total effect of bacterial inoculation**		H(7)=31.457,***		H(7)=26.125,***		H(7)=20.424,**		H(7)=43.475,***		H(7)=22.72,**	
**Total effect of microcystins**		H(2)=60.12,***		H(2)=65.882,***		H(2)=70.928,***		H(2)=41.256,***		H(2)=68.031,***	

Values are means of 4 replicates, and values preceded by ± are standard deviations. Differences between groups were considered statistically significant at a probability level of p≤0.05 using the Benjamini-hochberg false discovery rate correction. Means not sharing the same letter are significantly different. * = p<0.05; **= p<0.01; ***= p<0.001.

Table S3: Effects of bacterial inoculation and increasing concentrations of Microcystins on Electrolyte leakage (EL), and Malondialdehyde (MDA), ascorbic acid (AsA), and Polyphenols (GAE) in pea

Bacterial Inoculations	MC concentrations µg/L	Electrolyte leakage %	% Increase (+) or Decrease (-)	Malondialdehyde nmol.g^{-1} FW	% Increase (+) or Decrease (-)	Ascorbic acid content mg.100 g^{-1} FW	% Increase (+)	Polyphenols mg GAE.g^{-1}DW	% Increase (+) or Decrease (-)
**NI**	0	25.26±0.82i		319.98±8.67gh		10.73±0.84^{m}		14.78±0.01d	
	50	81.69±4.9b	56.43	419.39±7.98^{e}	31.07	11.83±0.57^{lm}	10.25	20.44±0.75bc	38.29
	100	87.27±3.28a	62.01	758.7±12.63a	137.11	13.31±0.63^{lm}	24.04	33.24±0.01a	124.9
**A**	0	16.74±1.04jk	-8.52	305.06±3.78hi	-4.66	14.47±3.23^{kl}	34.86	11.3±0.58f-h	-23.55
	50	59.48±4.48de	34.22	352.16±17.38f	10.06	16.56±1.98i-k	54.33	15.21±0.77d	2.91
	100	75.87±2.93c	50.61	642.83±13.32b	100.9	17.78±0.34g-j	65.7	22.22±1.38b	50.34
**B**	0	18.2±3.66jk	-7.06	289.84±10.84ij	-9.42	14.51±2.04kl	35.23	11.41±0.84f-h	-22.8
	50	63.12±3.93d	37.86	336.05±22.94fg	5.02	16.68±1.31i-k	55.45	15.06±1.37d	1.89
	100	75.99±5.33c	50.73	619.85±22.31c	93.72	17.75±0.5g-j	65.42	21.52±1.55b	45.6
**C**	0	20.27±4.57ij	-4.99	284.77±23.88j	-11	16.29±3.35jk	51.82	11.78±1.05fg	-20.3
	50	60.51±2.5de	35.25	346.65±17.82f	8.33	17.06±0.86h-k	58.99	14.46±1.33d	-2.17
	100	72.68±5.78c	47.42	621.58±17.8c	94.26	18.02±0.43f-j	67.94	18.98±2.3c	28.42
**AB**	0	15.86±1.78jk	-9.4	214.42±4.31l	-32.99	19.07±1.26d-i	77.73	11.28±1.25f-h	-23.68
	50	46.59±1.49g	21.33	260.84±16.66k	-18.48	20.05±1.64c-g	86.86	10.56±1.39f-h	-28.55
	100	55.59±8.17ef	30.33	483.2±9.31d	51.01	21.75±3.45b-d	102.7	14.93±2.02d	1.01
**AC**	0	16.59±2.94jk	-8.67	218.39±4.26l	-31.75	18.98±2.41e-j	76.89	9.61±2.09hi	-34.98
	50	46.21±1.83g	20.95	260.06±13.71k	-18.73	20.59±2.14c-f	91.89	11.61±0.32fg	-21.45
	100	52.18±3.43f	26.92	478.62±11.37d	49.58	21.28±1.63b-e	98.32	13.9±1.93de	-5.95
**BC**	0	16.88±2.72jk	-8.38	215.01±4.63l	-32.81	16.79±0.84i-k	56.48	10.16±2.13g-i	-31.26

	50	46.45±0.38g	21.19	261.1±12.24k	-18.4	18.64±1.31e-j	73.72	12.21±0.65ef	-17.39
	100	50.9±7.96fg	25.64	485.17±11.79d	51.63	19.74±4.53c-h	83.97	15.35±1.94d	3.86
**ABC**	0	12.77±2.63k	-12.49	121.38±0.06m	-62.07	22.34±0.39bc	108.2	7.49±0.15j	-49.32
	50	24.01±0.98i	-1.25	130.79±4.2m	-59.13	23.85±0.8b	122.27	8.48±0.16ij	-42.63
	100	31.83±0.77h	6.57	215.33±5.09l	-32.71	27.33±0.82a	154.71	11.99±0.001fg	-18.88
**Total effect of bacterial inoculation**		H(7)=23.95,**		H(7)=43.928,***		H(7)=66.303,***		H(7)=40.027,***	
**Total effect of microcystins**		H(2)=63.764,***		H(2)=45.201,***		H(2)=7.26,*		H(2)=39.19,***	

Values are means of 4 replicates, and values preceded by ± are standard deviations. Differences between groups were considered statistically significant at a probability level of p≤0.05 using the Benjamini-hochberg false discovery rate correction. Means not sharing the same letter are significantly different. * = p<0.05; **= p<0.01; ***= p<0.001.

Table S4: Effects of bacterial inoculation, increasing concentrations of Microcystins on total leaf phosphorus (TLP), total fruit phosphorus (TFP), and fruit minerals: Na, Ca, and K in pea

Bacterial Inoculations	MC concentrations µg/L	Total fruit phosphorous content mg.100g^{-1} DW	% Increase (+) or Decrease (-)	Total leaf phosphorous content mg.100g^{-1} DW	% Increase (+) or Decrease (-)	Fruit calcium (mg.100g^{-1} DW)	% Increase (+) or Decrease (-)	Fruit Potassium (mg.100g^{-1} DW)	% Increase (+) or Decrease (-)	Fruit Sodium (mg.100g-1 DW)	% Increase (+) or Decrease (-)
**NI**	0	78.85±3g		150.84±1.29e-g		42.54±0.77ef		220.18±4.11e-g		5.47±0.37h	
	50	59.68±0.44i	-24.31	114.05±6.12k-m	-24.39	32.17±3.83e-g	-24.38	175.1±1.31kl	-20.47	7.49±0.26cd	36.93
	100	54.23±4.8i	-31.22	67.1±2.57	-55.52	24.53±3.26jk	-42.34	162.66±10.84l	-26.12	10.52±2.48a	92.32

**A**	0	95.99±1.18d	21.74	159.21±12.51de	5.55	43.71±7.02l	2.75	225.55±15.47ef	2.44	3.86±0.27ij	-29.43
	50	72.4±1.23h	-8.18	120.13±9.14j-l	-20.36	39.36±2.68f-h	-7.48	200.98±7.07ij	-8.72	6.39±0.4e-g	16.82
	100	56.1±6.47i	-28.85	87.34±13.76n	-42.1	32±1.21jk	-24.78	180.37±1.41k	-18.08	8.84±0.57b	61.61
**B**	0	94.14±1.34de	19.39	158.59±5.17d-f	5.14	43.95±4.09d-f	3.31	214.05±23.45f-h	-2.78	4.07±0.1ij	-25.59
	50	76.62±2.82gh	-2.83	123.48±11.1i-k	-18.14	36.67±3.05h-j	-13.8	195.15±6.73j	-11.37	6.32±0.24e-h	15.54
	100	58.8±9.6i	-25.43	85.7±11.79n	-43.18	31.35±1.76k	-26.3	180.43±1.77k	-18.05	8.42±0.48b	53.93
**C**	0	94.94±0.48de	20.41	134.71±20.5hi	-10.69	49.04±3.47b-d	15.28	239.75±15.77cd	8.89	4.01±0.28ij	-26.69
	50	75.5±1.15gh	-4.25	127.82±10.39ij	-15.26	37.87±1.11g-i	-10.98	201.68±4.43h-j	-8.40	6.43±0.09ef	17.55
	100	55.33±5.82i	-29.83	84.66±3.91n	-43.87	32.06±0.88jk	-24.64	179.5±1.88k	-18.48	8.29±0.32bc	51.55
**AB**	0	123.05±4.41b	56.06	175.24±4.97bc	16.18	51.07±2.83bc	20.05	254.61±2.39b	15.64	3.31±0.41j	-39.49
	50	94.4±6.98de	19.72	146.49±1.06f-h	-2.88	43.12±4.67ef	1.36	223.1±0.9ef	1.33	5.55±0.21gh	1.46
	100	77.71±1.73gh	-1.45	109.18±9.76lm	-27.62	32.82±1.78i-k	-22.85	208.84±6.21g-i	-5.15	6.43±0.23ef	17.55
**AC**	0	120.49±3.33b	52.81	165.99±6.5cd	10.04	53.04±5.83b	24.68	252.07±7.58bc	14.48	3.43±0.14j	-37.29
	50	90.17±1.09ef	14.36	140.79±5.63gh	-6.66	46.31±7.53c-e	8.86	226.38±11.44ef	2.82	5.72±0.15f-h	4.57
	100	78.2±1.32g	-0.82	105.29±3.6m	-30.2	32.33±3.96jk	-24	207.39±8.92g-j	-5.81	6.97±0.47de	27.42
**BC**	0	123.39±5.6b	56.49	180.63±11.16b	19.75	53.57±6.84b	25.93	254.83±5.58b	15.74	3.46±0.22ij	-36.75

	50	98.34±5.37d	24.72	142.27±3.91gh	-5.68	41.4±1.73e-h	-2.68	217.76±11.47e-g	-1.10	5.59±0.32f-h	2.19
	100	74.07±2.89gh	-6.06	102.77±8.42m	-31.87	34.05±1.75i-k	-19.96	202.35±12.44h-j	-8.10	6.69±0.34de	22.3
**ABC**	0	140.15±1.68a	77.74	206.83±4.6a	37.12	77.65±1.64a	82.53	273.02±2.93a	24.00	2.05±0.26k	-62.52
	50	111.52±0.73c	41.43	171.94±1.67bc	13.99	53.79±1.46b	26.45	241.97±7.31bc	9.90	3.37±0.66j	-38.39
	100	85.05±4.49f	7.86	148.03±11.95e-g	-1.86	44.45±2.14d-f	4.49	227.98±4.52de	3.54	4.28±0.23i	-21.76
**Total effect of bacterial inoculation**		H(7)=38.682,***		H(7)=25.43,***		H(7)=28.368,***		H(7)=41.277,***		H(7)=29.599,***	
**Total effect of microcystins**		H(2)=50.021,***		H(2)=61.66,***		H(2)=53.162,***		H(2)=42.913,***		H(2)=59.308,***	

Values are means of 4 replicates, and values preceded by ± are standard deviations. Differences between groups were considered statistically significant at a probability level of p≤0.05 using the Benjamini-hochberg false discovery rate correction. Means not sharing the same letter are significantly different. * = p<0.05; **= p<0.01; ***= p<0.001.

Table S5: Effects of bacterial inoculation and increasing concentrations of Microcystins on sugars and proteins both in leaf and fruit in pea

**Bacterial Inoculations**	**MC treatment (µg.L-1)**	**Fruit soluble proteins (g.100g-1 FW)**	**% Increase (+) or Decrease (-)**	**Leaf soluble proteins (g.100g-1 FW)**	**% Increase (+) or Decrease (-)**	**Crude fruit proteins (g.100g-1 FW)**	**% Increase (+) or Decrease (-)**	**Crude leaf proteins (g.100g-1 FW)**	**% Increase (+) or Decrease (-)**	**Fruit soluble sugars (g.100g-1 FW)**	**% Increase (+) or Decrease (-)**	**Leaf soluble sugars (g.100g-1 FW)**	**% Increase (+) or Decrease (-)**
**NI**	0	5.27±0.45fg		4.93±0.04ef		10.86±0.24g		8.35±0.25h		8.04±0.83hi		6.2±0.12g	
	50	4.19±0.05ij	-20.49	3.4±0.09g	-31.03	7.73±0.38i	-28.82	6.53±0.01l	-21.8	6.39±0.5j	-20.52	9.6±0.19c	54.84

	100	2.35±0.2k	-55.41	2.07±0.12h	-58.01	5.29±0.8j	-51.29	3.87±0.32m	-53.65	4.19±0.36l	-47.89	13.47±0.06a	117.26
**A**	0	7.34±0.45c	39.28	6.03±0.2d	22.31	14.67±0.88d	35.08	11.95±0.23c	43.11	9.65±0.63d	20.02	5.07±0.11j	-18.23
	50	5.68±0.83ef	7.78	4.27±0.32f	-13.39	11.32±0.44fg	4.24	10.5±0.34f	25.75	8.39±0.28gh	4.35	7.4±0.14f	19.35
	100	3.7±0.53j	-29.79	2.12±0.96h	-57	8.38±0.47i	-22.84	7.21±0.18k	-13.65	5.06±0.19k	-37.06	11.05±0.1b	78.23
**B**	0	6.38±0.69e	21.06	6.02±0.33d	22.11	14.98±0.69d	37.94	12.11±0.09c	45.03	9.34±0.58d-f	16.17	5.06±0.1j	-18.39
	50	5.03±0.97f-h	-4.55	4.33±0.4ef	-12.17	11.48±1.26fg	5.71	10.23±0.3g	22.51	8.09±0.13hi	0.62	7.49±0.09f	20.81
	100	3.75±0.27j	-28.84	2.71±0.6gh	-45.03	9.62±0.35h	-11.42	7.29±0.21k	-12.69	5.26±0.46k	-34.58	11.14±0.08b	79.68
**C**	0	6.49±0.38de	23.15	6.18±0.59d	25.35	14.67±0.88d	35.08	12.15±0.14c	45.51	9.54±0.57de	18.66	5.05±0.17j	-18.55
	50	4.71±0.77g-i	-10.63	4.24±0.64f	-14	11.91±0.35fg	9.67	10.12±0.24g	21.2	7.47±0.16i	-7.09	7.38±0.12f	19.03
	100	3.93±0.48ij	-25.43	2.81±0.61gh	-43	8.24±0.36i	-24.13	7.14±0.25k	-14.49	5.1±0.6k	-36.57	11.2±0.09b	80.65
**AB**	0	9.87±1.48b	87.29	7.73±0.78c	56.8	20±0.22b	84.16	15.36±0.05b	83.95	11.97±0.26c	48.88	3.48±0.13l	-43.87
	50	7.21±0.16cd	36.81	6.46±0.73d	31.03	16.69±1.45c	53.68	11.55±0.15d	38.32	8.9±0.37e-g	10.7	5.65±0.04h	-8.87
	100	5.08±0.48fg	-3.61	5.04±0.59e	2.23	13.44±0.49e	23.76	8.17±0.2hi	-2.16	6.57±0.5j	-18.28	9.23±0.08d	48.87
**AC**	0	9.86±0.96b	87.1	7.71±0.73c	56.39	20.06±0.47b	84.71	15.49±0.1b	85.51	11.4±0.74c	41.79	3.61±0.08kl	-41.77
	50	7.44±0.09c	41.18	5.85±1.01d	18.66	15.67±0.59cd	44.29	11.43±0.12de	36.89	9.07±0.62d-f	12.81	5.42±0.18i	-12.58

	100	5.13±0.34fg	-2.66	4.52±0.5ef	-8.32	11.55±0.63fg	6.35	8.1±0.03hi	-2.99	6.39±0.37j	-20.52	9.21±0.12d	48.55
**BC**	0	9.07±0.38b	72.11	8.68±0.35b	76.06	19.38±0.45b	78.45	15.55±0.16b	86.23	11.34±0.41c	41.04	3.54±0.1l	-42.9
	50	7.58±0.18c	43.83	5.96±0.53d	20.89	15.62±0.87cd	43.83	11.46±0.12de	37.25	8.84±0.44fg	9.95	5.41±0.07i	-12.74
	100	4.25±0.74h-j	-19.35	4.94±0.44ef	0.2	12.14±0.97f	11.79	7.98±0.1i	-4.43	6.03±0.36j	-25	9.1±0.15d	46.77
**ABC**	0	11.89±0.32a	125.62	10.09±0.03a	104.67	22.26±0.32a	104.97	16.85±0.01a	101.8	17±0.59a	111.44	2.41±0.08m	-61.13
	50	9.39±0.15b	78.18	7.39±0.29c	49.9	16.56±1.8c	52.49	11.24±0.01e	34.61	13.02±0.12b	61.94	3.75±0.18k	-39.52
	100	7.66±0.06c	45.35	5.96±0.1d	20.89	13.25±0.1e	22.01	7.65±0.03j	-8.38	9.64±0.39d	19.9	7.67±0.13e	23.71
**Total effect of bacterial inoculation**		H(7)=47.583,***		H(7)=43.671,***		H(7)= 49.898,***		H(7)= 25.298,***		H(7)=35.237,***		H(7)=22.542,**	
**Total effect of microcystins**		H(2)=39.023,***		H(2)=44.005,***		H(2)=39.418,***		H(2)=65.537,***		H(2)=53.56,***		H(2)=69.912,***	

Values are means of 4 replicates, and values preceded by ± are standard deviations. Differences between groups were considered statistically significant at a probability level of p≤0.05 using the Benjamini-hochberg false discovery rate correction. Means not sharing the same letter are significantly different. * = p<0.05; **= p<0.01; ***= p<0.001.
